# Offset, Linear and Quadratic Scene-Change Artifact Comparison in Fourier-Transform Spectroscopy of a Target with Sharp Spectral Features

**DOI:** 10.3390/s26175326

**Published:** 2026-08-22

**Authors:** Kody A. Wilson, Michael L. Dexter, Benjamin F. Akers, Anthony L. Franz

**Affiliations:** Center for Technical Intelligence Studies and Research, Air Force Institute of Technology, 2950 Hobson Way, Fairborn, OH 45433, USA

**Keywords:** scene change artifact (SCA), smooth offset correction (SOC), Fourier-transform spectroscopy (FTS), hyperspectral imaging (HSI)

## Abstract

**Highlights:**

**What are the main findings?**

- Smooth offset correction effectively removed scene-change artifacts in targets with significant spectral structure during observations of time-varying scenes with Fourier-transform spectrometers.
- Following smooth offset correction, higher-order scene-change artifacts have minimal impact on spectral accuracy.

**What are the implications of the main findings?**

- Scene-change artifacts can be mitigated with minor modifications to traditional processing techniques and without modification of the data acquisition process.
- This approach enables Fourier-transform spectroscopy to accurately observe time-varying scenes with high spectral accuracy.

**Abstract:**

Observations of time-varying scenes using a Fourier-transform spectrometer can produce scene-change artifacts in the measured spectra. These artifacts typically appear as oscillations in the spectra and are often mistaken for noise. Interferogram-offset scene-change artifacts arise from an incorrect interferogram offset estimate. The error is Fourier-transformed and superimposed onto the measured spectrum. The recently developed smooth offset correction method removes these artifacts. Prior to this work, this correction method had only been applied to spectrally smooth targets. Concerns have been raised regarding its application to targets with structured spectra. In addition to the interferogram-offset scene-change artifacts, higher-order artifacts are predicted to be significant for structured spectra. Linear scene-change artifacts occur when a target moves with constant velocity through the field of view, and quadratic scene-change artifacts occur when a target moves with constant acceleration. These artifacts are largest near abrupt spectral transitions. Despite this, the higher-order artifacts remain relatively small and have minimal impact on spectral accuracy. We conclude that, after smooth offset correction, the remaining scene-change artifacts can be ignored when examining spectrally smooth targets such as notch filters.

## 1. Introduction

Scene-change artifacts (SCAs) appear in measured spectra when observing time-varying scenes using Fourier-transform spectroscopy (FTS). FTS instruments measure interferograms containing spectral information [1]. To measure an interferogram, a mirror must traverse a fixed distance, a sub-second to multi-minute process providing ample time for scene changes to occur [2]. Scenes with oscillating brightness, such as observing through a chopper wheel, produce a large SCA at a single wavenumber with smaller SCAs occurring at the harmonics [3,4,5]. Targets moving smoothly through the field of view (FOV), as well as targets with time-varying temperature, produce oscillations superimposed on the measured spectra [6,7,8]. The recombobulation correction method (RCM) and smooth offset correction (SOC) were recently developed to mitigate these SCAs. RCM mitigates SCAs caused by oscillating brightness through recombobulation of the target interferogram. SOC mitigates SCAs from both FOV and time-variable temperature scenes by improving interferogram-offset estimation. However, SOC has thus far only been applied to spectrally smooth targets, such as blackbodies. The SOC performance for targets with structured spectra has not been evaluated. Furthermore, higher-order SCAs, which are not mitigated by SOC, are predicted for such targets [9]. This paper will analyze the following three types of SCAs: interferogram-offset, linear, and quadratic SCAs.

Interferogram-offset SCAs arise from brightness changes. These artifacts appear as oscillations throughout the measured spectra, including regions outside instrument response band [6]. These SCAs exhibit relatively uniform magnitude across the measured spectra but increase in amplitude near zero wavenumber. One of the initial steps in traditional FTS processing is subtraction of the interferogram offset. Traditional FTS processing assumes a constant interferogram offset, which is estimated by averaging the measured interferogram [10]. However, this assumption does not account for changes in scene brightness that produce a variable interferogram offset. Consequently, an error arises between the variable offset and the constant offset estimate. This error is Fourier-transformed and superimposed onto the measured spectra. These SCAs are often mistaken for noise in the measured spectra [11,12,13]. SOC employs a variable interferogram-offset estimate, thereby eliminating the error and the associated SCAs.

Linear SCAs appear in the imaginary component of the spectra and occur when there is a linear transition between targets, such as a target moving through the FOV with constant speed [6,9]. Linear SCAs are expected in regions of the spectrum where there are sudden changes with respect to wavenumber. Since linear SCAs occur in the imaginary component of the spectrum they have no effect on the real component of the spectrum without phase correction or complex calibration. When phase correction is used the linear SCAs can affect the real component of the measured spectrum.

Quadratic SCAs appear in the real component of the spectra and occur when there is a quadratic transition between targets, such as those produced by a target moving through the FOV with constant acceleration [6,9]. Similar to linear SCAs, quadratic SCAs are expected to be most significant near abrupt spectral changes with respect to wavenumber. The analysis presented in this paper is limited to these three SCA types. More complex motion through the FOV can produce higher-order terms that may be analyzed using the methodology presented in this paper. More complex motion, where the position of the target is represented by a high-order polynomial will produce corresponding SCA terms for each degree of polynomial required to represent the target’s position. Each additional SCA term is derived through the same process as the linear term derived in Section 2.1. Odd order terms are expected to produce imaginary SCAs, while even order terms produce real SCAs.

First, this paper presents the theoretical framework for these three SCA types. This theoretical development also demonstrates how SOC eliminates the interferogram-offset SCAs. The methodology for simulating interferogram measurements with linear and quadratic scene changes is then described. Linear and quadratic scene changes are then simulated, and each of the three SCA types is analyzed along with the expected improvement provided by SOC for interferogram-offset SCAs. These simulations approximate the conditions used in the subsequent experiment.

After establishing the theoretical framework, the experimental setup is described. The experiment involved translating a notch filter in front of a blackbody while the scene was measured using an FTS hyperspectral imager (HSI). Moving the filter at constant velocity produced linear SCAs, whereas motion with constant acceleration produced quadratic SCAs. Interferogram-offset SCAs were observable and mitigated using SOC. Linear SCAs were observable in the imaginary component of the spectrum. However, they had little impact on the real component. Quadratic SCAs minimally affected the spectrum. The experiment was designed to maximize the effects of linear and quadratic SCAs. Despite these conditions, the higher-order SCAs had negligible effect on the measured spectrum. These results suggest that, once the interferogram-offset SCAs are mitigated using SOC, the remaining FOV SCAs have negligible impact on the overall accuracy of FTS spectra.

## 2. Theory and Simulation

### 2.1. Fourier-Transform Spectroscopy and Scene-Change Artifact Theory

The measurement scenario considered includes observing a target while a second target enters the FOV of an FTS instrument. The FTS interferometer splits the incoming light into two paths, recombines the beams, and measures the resulting interference signal. The measured interferogram is the measured signal versus optical path difference (OPD) between the two paths [14]. The OPD is varied using a movable mirror on one of the paths. While the mirror scans through different OPDs, the observed scene changes. The observed radiance at each OPD is the weighted average of the spectra from the two targets, with weighs determined by the fraction of the FOV filled by each target. The spectrum observed by the FTS as a function of OPD is given by the following:

(1) $$L\left(x,\nu \right)=\left[1-s(x)\right]L_{1}\left(\nu \right)+s(x)L_{2}\left(\nu \right)$$

where $L_{1}(x)$ is the spectrum of target 1, $L_{2}(x)$ is the spectrum of target 2, s(x) is the scene function, and x is the OPD. The scene function represents the fraction of the FOV filled by target 2 [6]. Because OPD is linearly proportional to time, it is convenient to express the scene function in terms of OPD rather than time. If the scene function is represented by a polynomial, each term produces a corresponding SCA term that is superimposed on the spectrum. The measured spectrum is given by the following:

(2) $$L_{m}\left(\nu \right)=L_{ZPD}\left(\nu \right)+L_{Offset,SCA}\left(\nu \right)+L_{Linear,SCA}\left(\nu \right)+L_{Quadratic,SCA}\left(\nu \right)+\cdots$$

where $L_{ZPD}\left(\nu \right)$ is the spectrum of the scene at the conditions occurring at zero path difference (ZPD), $L_{Offset,SCA}\left(\nu \right)$ is the SCA term arising from incorrect interferogram offset estimation, $L_{Linear,SCA}(\nu )$ is the linear SCA term, and $L_{Quadratic,SCA}(\nu )$ is the quadratic SCA term, and ν is the wavenumber. The remainder of this section examines each term individually.

The most prevalent and easily mitigated source of SCAs is the interferogram-offset term. To understand its origin, first consider the measured interferogram for a static target, given by the following:

(3) $$I_{m}\left(x\right)=\left[I\left(x\right)+I_{o}\right]a(x)$$

where $I_{m}(x)$ is the measured interferogram, $I\left(x\right)$ is the interferogram without offset, and $I_{0}$ is the interferogram offset [15]. Lastly, a(x) represents the truncation or apodization function. In this work, the measured interferogram was truncated near the maximum optical path difference (MOPD) and no apodization was applied. The term “interferogram” is commonly used to refer to both $I_{m}(x)$ and I(x) [16]. This may explain why previous authors neglected the interferogram-offset SCAs in their SCA predictions [17,18]. The interferogram offset is often referred to as the DC component because, under normal operating conditions, it is constant and easily removed. The interferogram offset is typically estimated by averaging the measured interferogram [19]. Subtracting this estimate and Fourier-transforming the result yields the measured spectrum, given by the following:

(4) $$L_{m}\left(\nu \right)=I\left\{\left[I_{m}\left(x\right)-I_{o,est}\right]a(x)\right\}$$

where I represents the Fourier transform, and $I_{o,est}$ is the estimated interferogram offset [20]. In practice, the Fourier-transform is typically computed with a fast Fourier transform (FFT) [21]. In this work, MATLAB R2024’s, fft was used to compute the FFT [22]. The constant offset estimate performs well under static conditions but produces errors when the scene changes within the FOV. Fourier transforming this error yields the interferogram offset SCAs, given by the following:

(5) $$L_{Offset,SCA}\left(\nu \right)=I\left\{\left[I_{o}\left(x\right)-I_{o,est}\right]a(x)\right\}$$

where $I_{o}(x)$ is the variable interferogram offset. When SCAs are not present, the truncation function limits spectral resolution [23]. When SCAs are present, the SCA oscillation frequencies are also determined by the truncation function. Interferogram-offset SCAs occur for all scene changes and are easily mitigated with SOC [6]. SOC approximates the variable interferogram offset using locally weighted scatterplot smoothing (LOWESS) [24]. Subtracting an accurate interferogram offset eliminates the estimation error and the associated interferogram-offset SCAs.

Next, the linear scene change is examined. At the beginning of interferogram collection, the FOV is filled by target 1, and at the end of the collection the FOV is filled by target 2. The associated scene function is given by the following:

(6) $$s\left(x\right)=\frac{x+\frac{x_{a}}{2}}{x_{a}}$$

where $x_{a}$ is the width of the interferogram. For a two-sided symmetric interferogram, the width is twice the MOPD. Substituting the linear scene function into Equation (1) yields the spectrum as a function of OPD:

(7) $$L\left(x,\nu \right)=\left(1-\frac{x+\frac{x_{a}}{2}}{x_{a}}\right)L_{1}\left(\nu \right)+\frac{x+\frac{x_{a}}{2}}{x_{a}}L_{2}\left(\nu \right).$$

The associated measured interferogram is given by the following:

(8) $$I_{m}\left(x\right)=\left\{\left(1-\frac{x+\frac{x_{a}}{2}}{x_{a}}\right)\left[I_{1}\left(x\right)+I_{o,1}\right]+\frac{x+\frac{x_{a}}{2}}{x_{a}}\left[I_{2}\left(x\right)+I_{o,2}\right]\right\}a\left(x\right)$$

where $I_{1}(x)$ and $I_{2}(x)$ are the interferograms without offset for static targets 1 and 2. Similarly, $I_{o,1}$ and $I_{o,1}$ are the interferogram offsets for static targets 1 and 2. Making the substitutions $I_{1}\left(x\right)=I^{-1}[L_{1}(\nu )]$ and $I_{2}\left(x\right)=I^{-1}\left[L_{2}\left(\nu \right)\right]$ and then grouping terms yields the following:

(9) $$I_{m}\left(x\right)=\left\{\begin{array}{c} I^{-1}\left[\frac{L_{1}\left(\nu \right)+L_{2}\left(\nu \right)}{2}\right]+\frac{x}{x_{a}}I^{-1}\left[L_{2}\left(\nu \right)-L_{1}\left(\nu \right)\right]\\ +\frac{x}{x_{a}}\left(I_{o,2}-I_{o,1}\right)+\frac{I_{o,2}-I_{o,1}}{2} \end{array}\right\}a\left(x\right)$$

where $I^{-1}$ is the inverse Fourier transform. The last term in the large, bracketed expression is removed in traditional processing. The second to last term is associated with the interferogram-offset SCAs and is removed using SOC. The first and second terms are Fourier-transformed and yield the ZPD spectrum and the linear SCA term. In this case, the ZPD spectrum is the average of the initial and final spectra modified due to the truncation function:

(10) $$L_{ZPD}\left(\nu \right)=\frac{L_{1}\left(\nu \right)+L_{2}(\nu )}{2}⊛A\left(\nu \right)$$

where A(ν) is the Fourier transform of the truncation function and ⊛ represents convolution [25]. For scene changes that are not symmetric about ZPD, the ZPD spectrum becomes the weighted average of the two measured spectra, with the weighs determined by the fraction of the FOV filled by each target.

The Fourier transform of the second term in Equation (9) yields linear SCAs. Multiplication by x in the interferogram domain leads to a derivative in the spectral domain. The linear SCA term, previously derived by Kick, becomes the following [9]:

(11) $$L_{Linear,SCA}\left(\nu \right)=i\frac{1}{2\pi x_{a}}\frac{\partial }{\partial \nu }\left[L_{2}\left(\nu \right)-L_{1}\left(\nu \right)\right]⊛A\left(\nu \right)$$

The derivative amplifies the linear SCA term when spectra contain structure. Since the linear SCA term is imaginary, it can typically be ignored. It affects the real component of the spectrum only when phase correction is applied [26]. Even when phase correction is applied, only a small part of the linear SCA contaminates the real component of the spectrum.

The same procedure can be used to derive the quadratic SCA term, given by the following [9]:

(12) $$L_{Quadratic,SCA}\left(\nu \right)=-\frac{1}{4\pi^{2}x_{a}^{2}}\frac{\partial^{2}}{\partial \nu^{2}}\left[2L_{1}\left(\nu \right)-4L_{ZPD}\left(\nu \right)+2L_{2}\right]⊛A\left(\nu \right)$$

Unlike the linear term, the quadratic term appears in the real component of the spectra. Similar to the linear SCAs, the derivative suggests that this term becomes larger when the spectrum contains structure. Higher-order terms could be examined, but it is important to note that these SCA terms arise from the scene function. If the scene function does not include higher-order terms, the associated SCA terms will not be present. For example, if the scene changes are linear, then quadratic SCAs are not expected. Higher-order SCA terms require higher-order scene functions, corresponding to more complex motion through the FOV.

### 2.2. Simulate Scene-Change Artifacts

**Figure 1:**
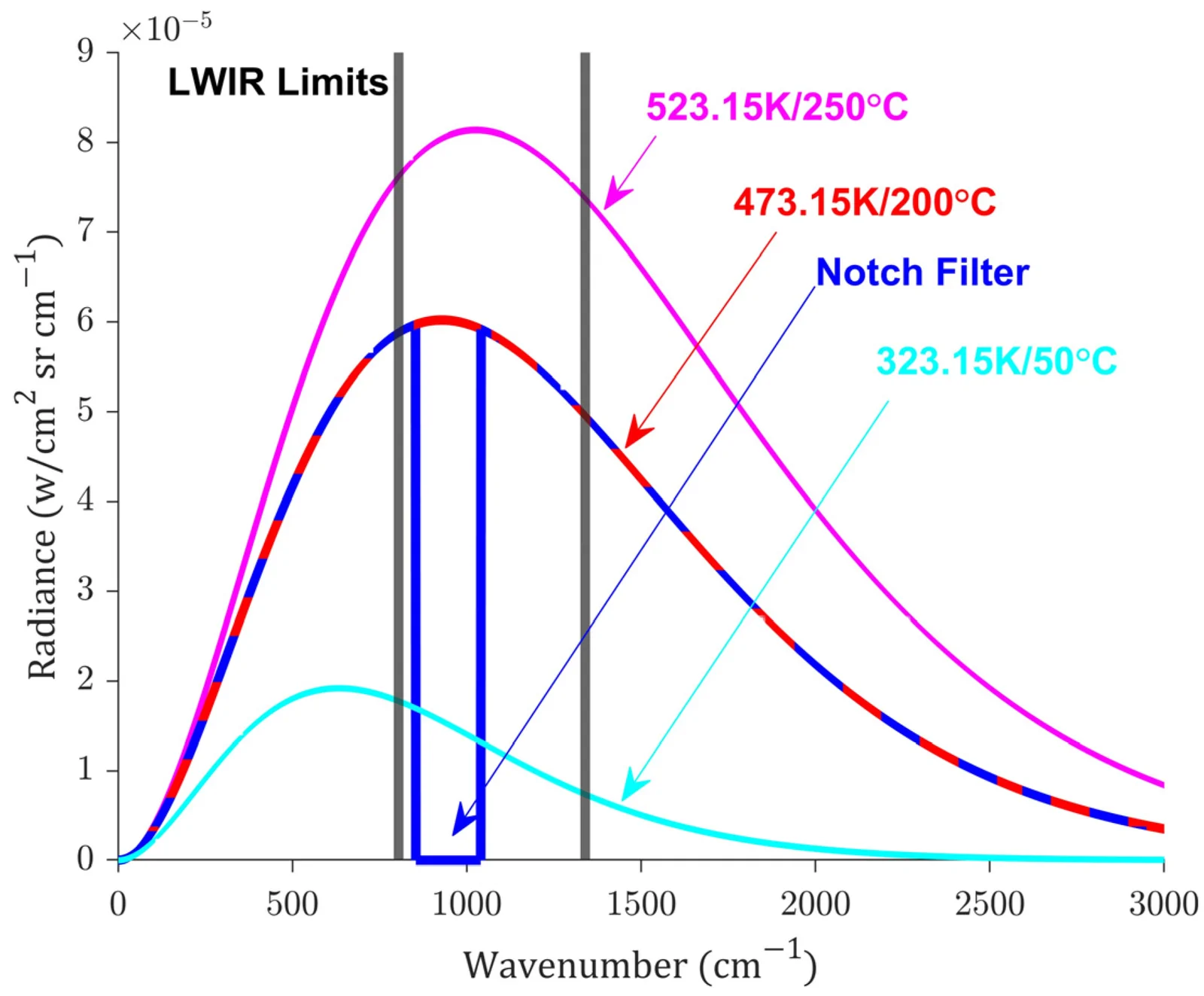
Simulated spectra used to model the FOV scene change. The red curve represents the spectrum of the blackbody target. The blue curve represents the spectrum of the blackbody observed through the notch filter. The magenta and cyan curves represent the spectra of the hot and cold blackbodies used for calibration. The vertical black lines indicate the longwave infrared (LWIR) calibration gain limits.

Simulations are used to examine the effect of the SCAs on the measured spectrum. The simulations begin by assuming a blackbody target with a spectrum given by the following:

(13) $$L\left(\nu ,T\right)=\frac{2{hc}^{2}\nu^{3}}{e^{\frac{hc\nu }{kT}}-1},$$

where T is the temperature, h is Planck’s constant, c is the speed of light, and k is Boltzmann’s constant [27]. The second spectrum represents a blackbody observed through a notch filter. The notch filter introduces spectral structure, and the scene change can be reproduced experimentally by translating the filter through the FOV. The second spectrum is given by the following:

(14) $$L_{2}\left(\nu ,T\right)=T_{filter}(\nu )L\left(\nu ,T\right)$$

where $T_{filter}\left(\nu \right)$ is the transmission function of the notch filter. The simulated notch filter transmission is 1 outside the notch and 0 within the notch. The notch filter has a full width of 186 cm^−1^ and is centered at 947 cm^−1^, matching the notch filter used in the experiment. Two additional blackbody spectra were simulated at different temperatures for calibration purposes. The four simulated spectra are shown in Figure 1.

After the spectra are simulated, the detector response is applied using the following:

(15) $$L_{uncal}\left(\nu \right)=G\left(\nu \right)[L\left(\nu \right)+O\left(\nu \right)]$$

where $L_{uncal}(\nu )$ is the uncalibrated spectrum, G is the calibration gain, and O is the calibration offset [28]. For these simulations, the gain was 1 within the LWIR region and 0 outside, while the offset was set to 0 everywhere. The uncalibrated spectrum is mirrored about zero wavenumber. The original and mirrored spectra are then added together. Taking half the inverse Fourier transform of the sum yields the simulated interferogram without offset [29]:

(16) $$I\left(x\right)=\frac{1}{2}I^{-1}\left[L_{uncal}\left(\nu \right)+L_{uncal}(-\nu )\right]$$

The inverse Fourier transformed is computed using an inverse fast Fourier transform (IFFT) in MATLAB R2024b [30]. The simulated interferogram offset is computed as follows:

(17) $$I_{o}\left(x\right)=I(0)$$

Substituting Equations (16) and (17) into Equation (3) yields the simulated measured interferogram. This process is repeated for each target spectrum.

The next step is to determine the measured interferogram during the FOV scene change. The measured interferogram is the weighted sum of the interferograms from the two targets, given by the following:

(18) $$I_{m}\left(x\right)=\left\{\left[1-s\left(x\right)\right]I_{1}\left(x\right)+s\left(x\right)I_{2}\left(x\right)\right\}a(x)$$

where $I_{1}\left(x\right)$ and $I_{2}\left(x\right)$ are the measured interferograms corresponding to target 1 and target 2. Note that this expression is the generalized form of Equation (8). The scene function is represented as either a linear or quadratic polynomial depending on the scene change considered. More complicated scene changes can be analyzed using the same procedure. However, this paper is limited to linear and quadratic scene changes.

Once the simulated measured interferogram is obtained, the associated measured spectrum must be determined. This process is the same for simulation and experiment. Using Equation (4), the measured spectra are converted into raw spectra. For the scene-change interferogram, the process was completed with and without SOC for comparison. The two raw blackbody spectra are used to estimate the calibration gain and offset using the following:

(19) $$G^{*}\left(\nu \right)=\frac{L_{hot,raw}\left(\nu \right)-L_{cold,raw}\left(\nu \right)}{L_{hot}\left(\nu \right)-L_{cold}(\nu )}$$

where $L_{hot,raw}\left(\nu \right)$ and $L_{cold,raw}\left(\nu \right)$ are the raw spectra of the hot and cold blackbodies, while $L_{hot}\left(\nu \right)$ and $L_{cold}\left(\nu \right)$ are the truth spectra of the hot and cold blackbodies [31,32]. The calibration offset is estimated using the following:

(20) $$O^{*}\left(\nu \right)=\frac{L_{cold,raw}\left(\nu \right)L_{hot}\left(\nu \right)-L_{hot,raw}\left(\nu \right)L_{cold}\left(\nu \right)}{L_{hot,raw}\left(\nu \right)-L_{cold,raw}(\nu )}$$

Solving Equation (15) for the target spectrum and using the estimated gain and offset, the calibrated spectrum is calculated using the following:

(21) $$L_{cal}=\frac{L_{raw}\left(\nu \right)}{G^{*}\left(\nu \right)}-O^{*}\left(\nu \right)$$

This may appear to be an unnecessary step, given that the simulation assumes a gain of 1 within the instrument response region and zero outside. However, including the calibration step improves overall simulation accuracy. The measured interferograms are truncated at the MOPD. This truncation causes oscillations near the edge of the response region for the blackbody and target raw spectra. These oscillations then appear in the estimated gain $G^{*}$, which in turn reduces the apparent oscillations in the target’s calibrated spectrum. This only reduces oscillations near the LWIR boundary and does not reduce oscillations near the notch filter boundary. Estimating the calibration gain and offset increases the accuracy of the simulated calibrated spectra relative to the truth spectra. Furthermore, the simulated calibrated spectra more closely match the experimental calibrated spectra. By adjusting the scene function in Equation (18), the same process can be used to simulate linear or quadratic scene changes.

### 2.3. Interferogram-Offset Estimation Error and Smooth Offset Correction

**Figure 2:**
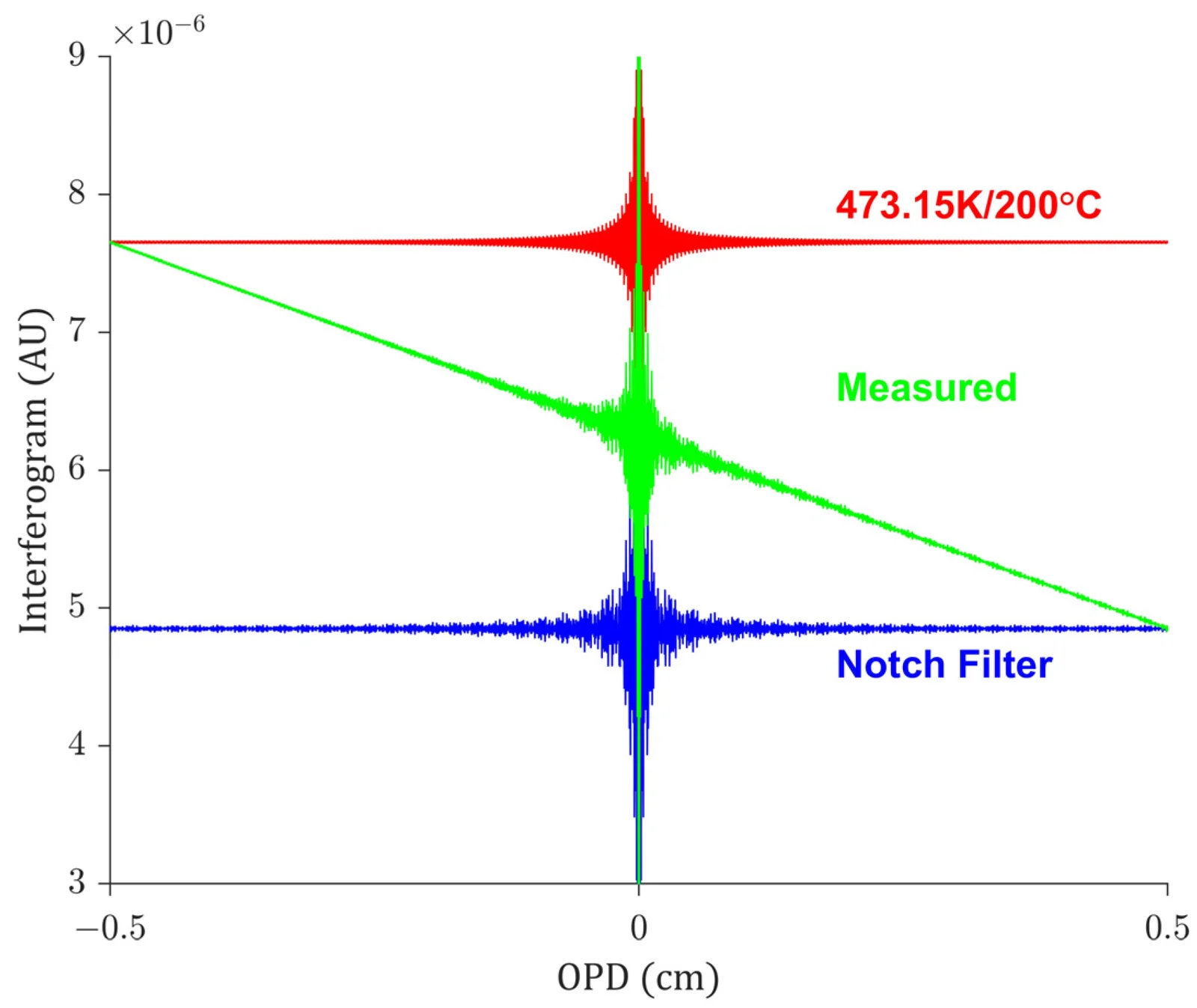
Simulated interferogram measurements for a linear scene change. The green curve represents the measured interferogram for the linear scene change. The red curve represents the measured interferogram for the unobscured blackbody, while the blue curve represents the measured interferogram for the blackbody observed through the notch filter.

Interferogram-offset SCAs are examined first. These SCAs are present in both FOV scene changes. A linear scene change was simulated following the procedure described in Section 2.2. The simulated interferogram measurements are shown in Figure 2.

**Figure 3:**
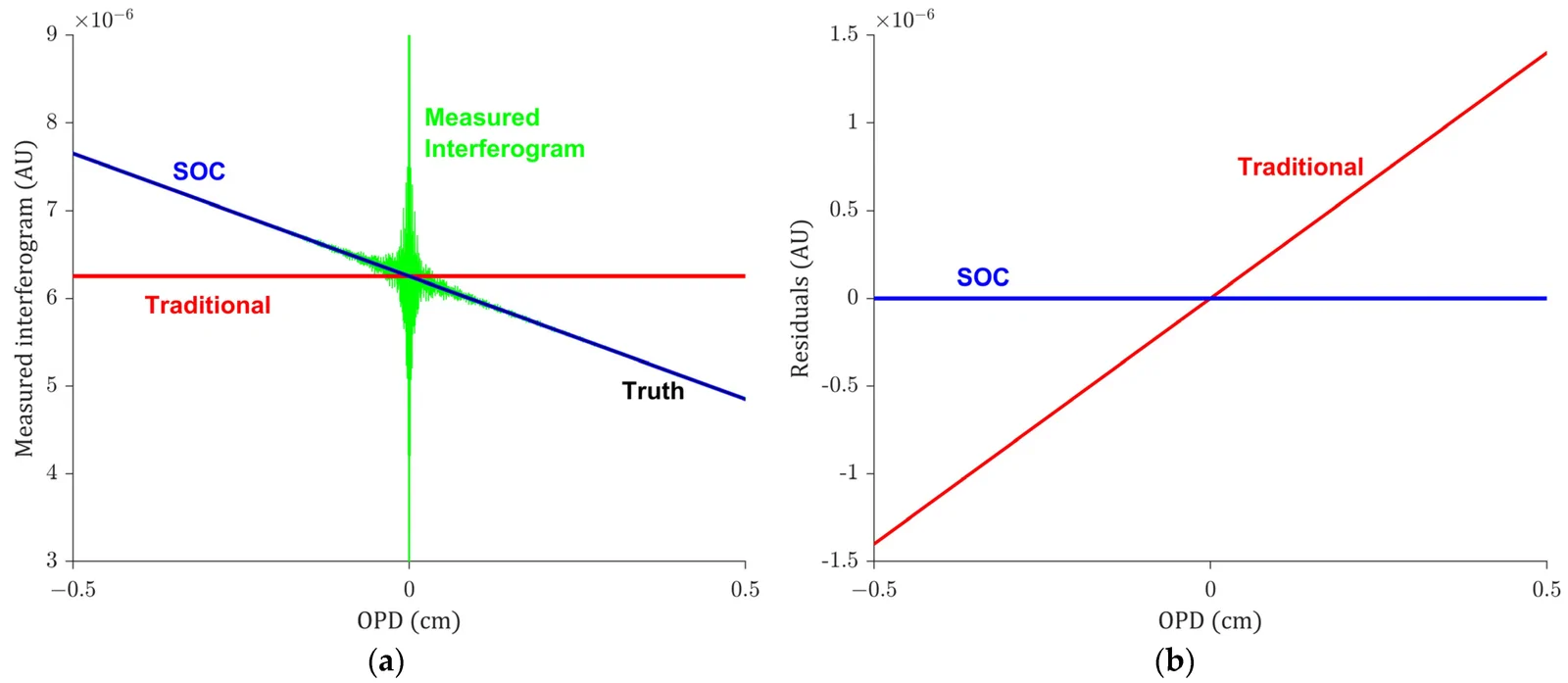
(**a**) Interferogram offset estimates using the traditional method and SOC. The green curve represents the measured interferogram from Figure 2. The red curve represents the traditional interferogram-offset estimate. The blue curve represents the SOC interferogram-offset estimate. The black curve represents the interferogram-offset truth. (**b**) Residuals between the interferogram-offset estimates and the offset truth. The red curve represents the error between the traditional interferogram-offset estimate and truth. The blue curve represents the error between the SOC interferogram-offset estimate and truth.

The linear variation in the interferogram offset gives the target’s measured interferogram its approximately linear appearance. The traditional approach estimates the interferogram offset by averaging the measured interferogram. This leads to significant errors between the true interferogram offset and its estimate, shown in red in Figure 3. The SOC estimate was computed using MATLAB R2024b’s smoothdata function with the LOWESS option [33]. Applying SOC yields a more accurate estimate of the interferogram offset, shown in blue in Figure 3.

Typically, the largest residuals occur near ZPD. A smoothing window size of approximately 0.02 cm was used for Figure 3. The residuals near ZPD can be reduced by increasing the window size, but large window sizes can reduce interferogram offset accuracy for rapid scene changes. The window size can be reduced to 0.005 cm before the error near ZPD reaches 1%. This provides a wide range of SOC window sizes that can be used to accommodate the expected scene change.

**Figure 4:**
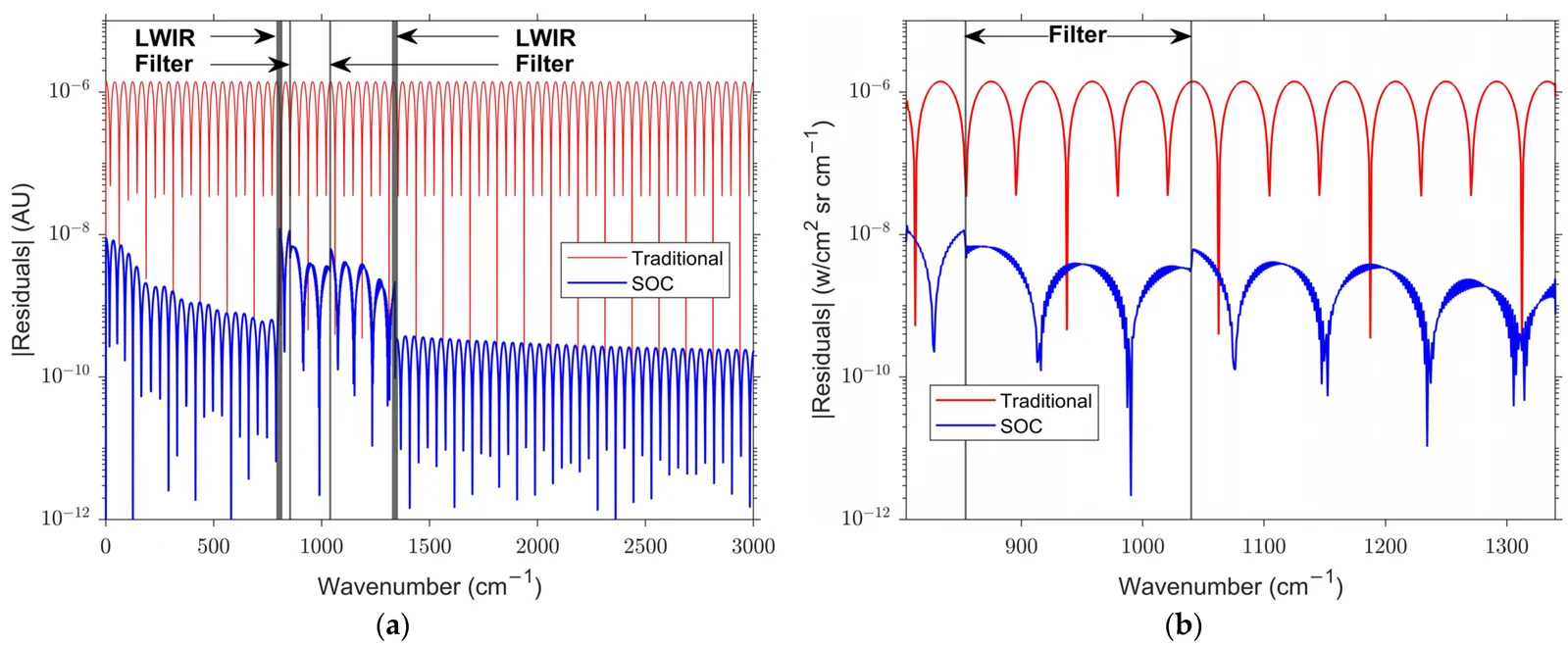
(**a**) Interferogram-offset SCA in the raw spectrum. The red curve represents the interferogram-offset SCA using the traditional method. The blue curve represents interferogram-offset SCA using SOC. The interferogram-offset SCA is the Fourier-transform of the interferogram-offset estimate error. The LWIR instrument and notch filter cutoffs are indicated in black. (**b**) Interferogram-offset SCAs in the calibrated spectrum. The red curve represents the interferogram-offset SCA using the traditional method. The blue curve represents the interferogram-offset SCA using SOC. The notch filter cutoffs are indicated in black. The interferogram-offset SCAs with SOC are near zero for both raw and calibrated spectra. Only the magnitude of the real component of the residual is displayed.

The Fourier transform of the estimation error yields the interferogram-offset SCAs. The traditional method leaves a larger error and SCAs, while SOC reduces the SCAs, as shown in Figure 4.

**Figure 5:**
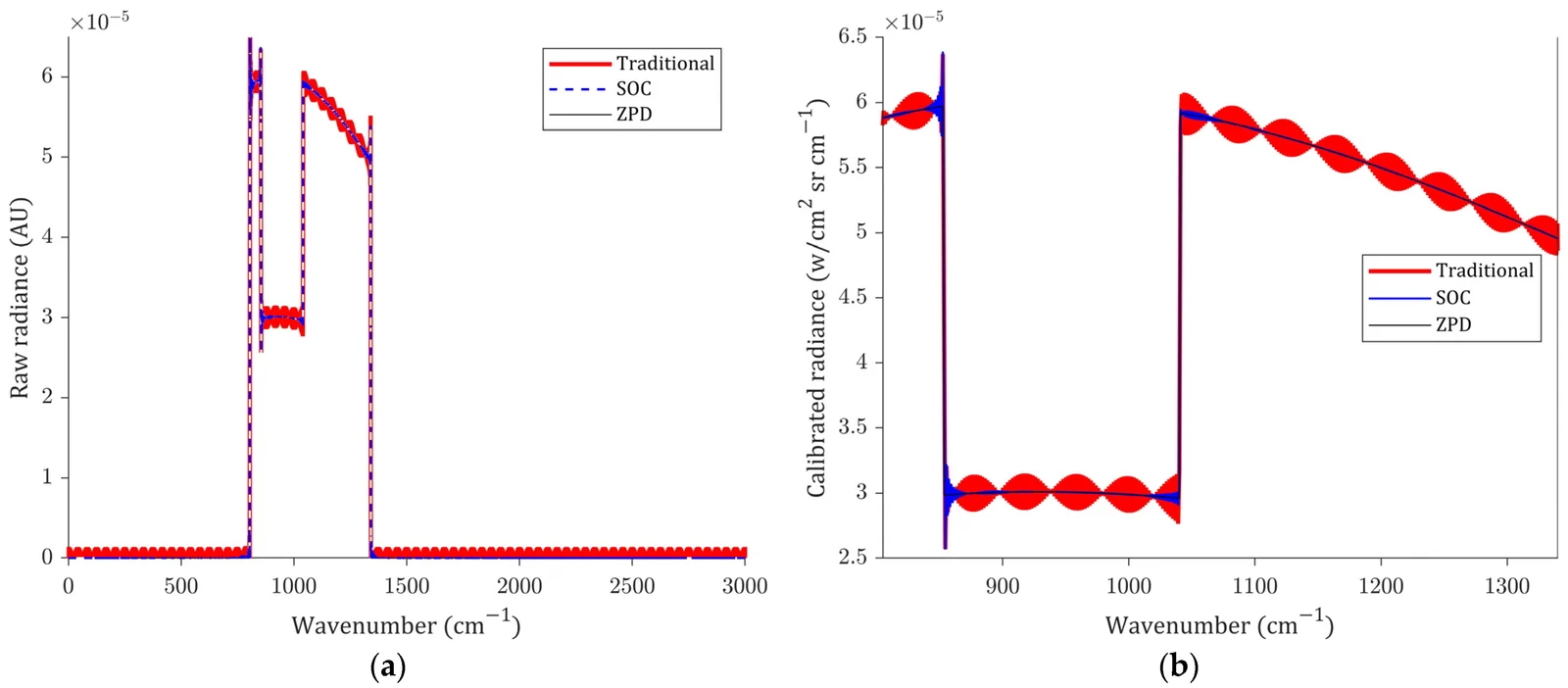
(**a**) Simulated raw spectra; (**b**) simulated calibrated spectra for a linear scene change. The red curves represent the traditionally processed spectra with SCAs. The blue curves represent the spectra processed with SOC. The black curves represent the ZPD spectra truth. The SOC spectrum is indistinguishable from the ZPD spectrum.

Figure 4 shows that SOC successfully mitigates SCAs. Figure 5 shows SCAs superimposed on the measured spectrum before and after SOC. The measured spectrum resembles the spectrum at ZPD. For the symmetric linear scene change, depicted in Figure 3, the ZPD spectrum is the average of the two target spectra, as given by Equation (10). A useful property of interferogram-offset SCAs is that they occur outside the instrument response region, as shown in Figure 4a and Figure 5a. Since the expected signal outside the response region is zero, any signal there can be attributed to SCAs and be used to estimate their magnitude.

These results for structured spectra match previous expectation from spectra without structure [6]. Additionally, note the error near the notch filter’s cutoff wavenumber, which appears in both the traditional and SOC spectra. This error arises from Gibbs phenomenon and truncation of the interferogram, rather than SCAs, and is examined more closely in Section 2.4 [34]. After SOC, Gibbs phenomenon is the dominant source of error. The normalized root mean square error (NRMSE) is used to quantify the error and compare before and after SOC. NRMSE is calculated using the following:

(22) $$\mathrm{NRMSE}=\frac{1}{n^{2}\{\max\left[L_{\mathrm{ZPD}}(\nu )\right]-\min\left[L_{\mathrm{ZPD}}(\nu )\right]\}}\sqrt{\sum_{i=1}^{n}{\left\{Re[L_{cal}(\nu_{i})]-L_{\mathrm{ZPD}}\left(\nu_{i}\right)\right\}}^{2}},$$

where n is the number of spectral bins, $L_{\mathrm{ZPD}}$ is the truth spectrum at ZPD, and $L_{cal}$ is the calibrated spectrum [35,36]. The first scenario listed in Table 1 corresponds to a notch filter centered at 947 $cm^{-1}$ with a bandwidth of 186 $cm^{-1}$, matching the filter used in the experiment. Additionally, Table 1 summarizes the NRMSE for different filter bandwidths and filter transmissions. Lastly, no scene change was simulated as a control.

Smaller filter bandwidths and higher transmission were associated with reduced SCAs. This is expected because these scenarios exhibit smaller differences in interferogram offset between the target and the target observed through the filter. For each scene change, SOC significantly reduces the error. For no scene change, SOC increases the error. In the no scene-change scenario, the interferogram offset is constant. The traditional constant offset assumption yields a more accurate estimate than the SOC variable offset estimate for this specific case. Despite this, the error from the incorrect estimate is still significantly smaller than the error from truncating the interferogram.

### 2.4. Linear SCAs and Phase Correction

**Figure 6:**
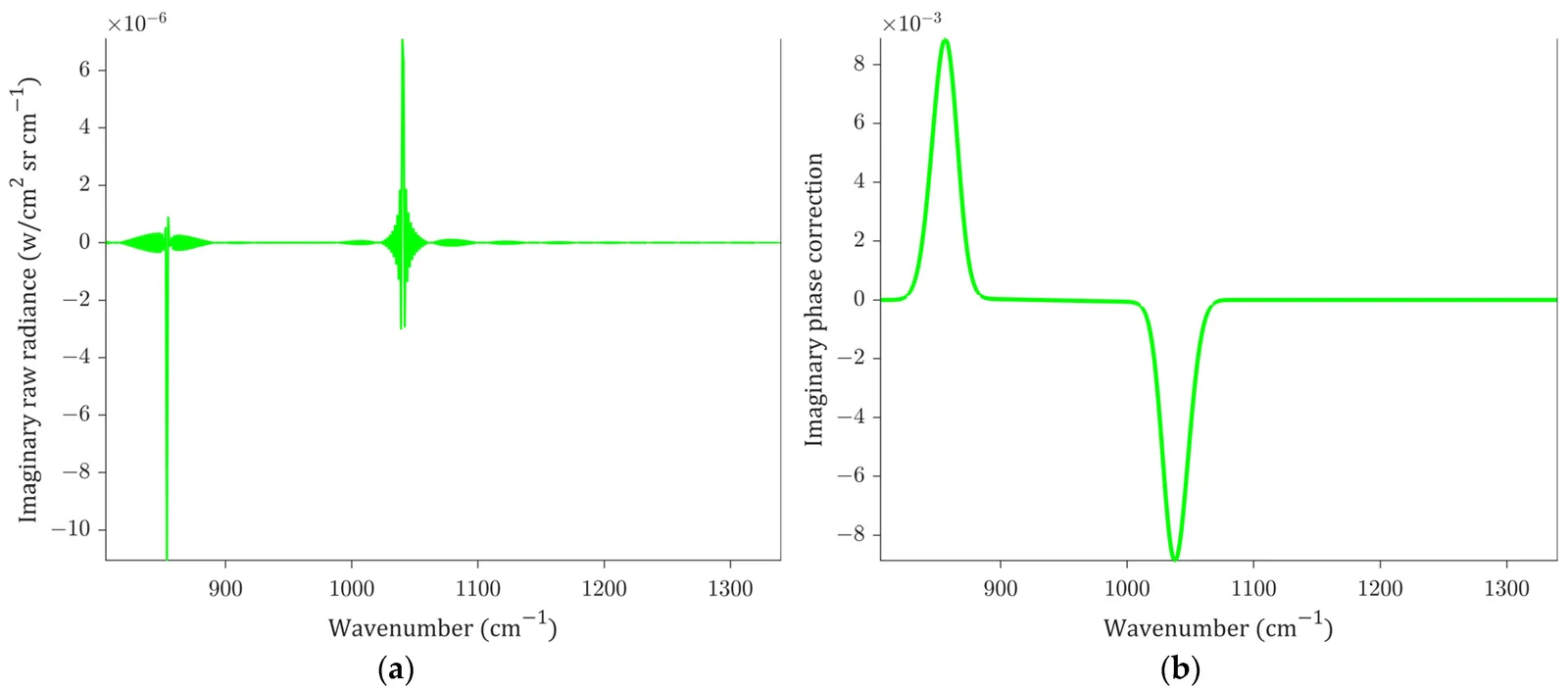
(**a**) Imaginary component of the raw SCAs for a linear scene change given by Equation (11). The imaginary SCAs increase near the notch filter’s cutoff wavenumbers, where the derivative is large. (**b**) Imaginary component of the phase correction. For most of the spectrum, it remains near zero with large increases noticeable near the filter cutoff wavenumbers.

Linear SCAs occur when the scene function includes a linear term such as a target moving with constant velocity into the FOV. Equation (11) predicts that linear SCAs appear in the imaginary component of the spectrum. Figure 6a shows the imaginary component of the calibrated spectrum for the measured interferogram presented in Figure 2. Without phase correction, these imaginary SCAs are discarded. The phase corrected spectrum is determined using the following:

(23) $$L_{raw,PC}\left(\nu \right)=[L_{raw}\left(\nu \right)]e^{-i\theta \left(\nu \right)}$$

where $e^{-i\theta }$ is the phase correction and θ is the phase angle. The phase angle is defined as the angle between the imaginary and real component of the raw spectra and is calculated at each wavenumber [26,27]. The phase-corrected raw spectrum is subsequently processed using the same methodology applied to the raw spectrum without phase correction. Although, no phase angle is present in this simulation; the imaginary SCAs influence the phase correction calculation, as shown in Figure 6b.

Equation (23) shows that the effect of linear SCAs on the real component of the raw spectrum is given by the product of Figure 6a,b. An interesting difference is that the imaginary phase component is much smoother despite being related to the rapid oscillation in the spectrum. This is because the default phase calculation uses a Gaussian apodization function while the default raw spectrum calculation uses a truncation function. The Gaussian function is used to prevent ringing in the phase calculation [27]. Adjusting either default is possible but with little change to linear SCA effect on accuracy.

**Figure 7:**
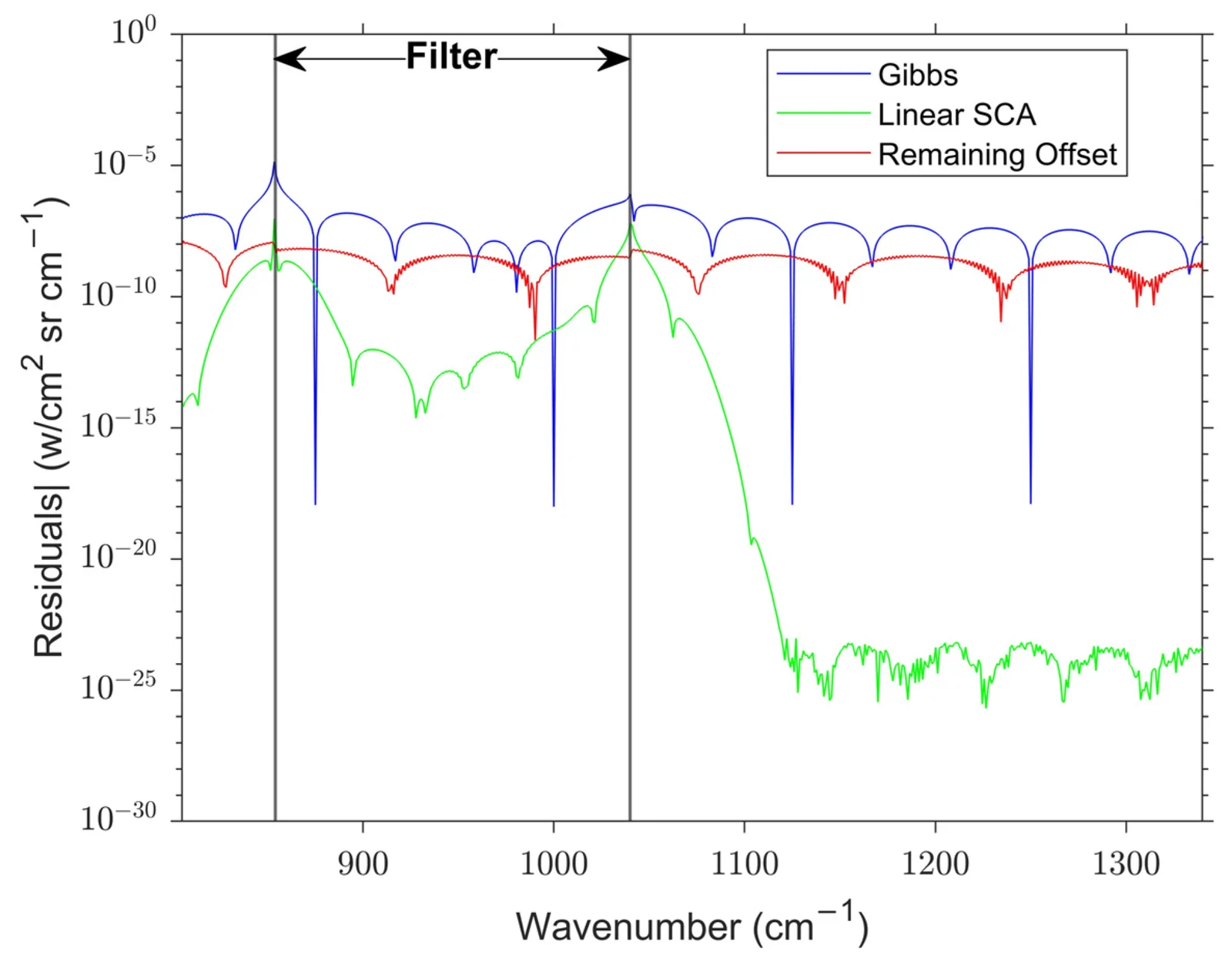
Comparison of simulated error sources for a linear scene change. The blue curve represents the error from the Gibbs phenomenon. The green curve represents the error from the linear SCAs. The red curve represents the error from interferogram-offset SCAs after SOC. The notch filter cutoffs are indicated in black. The Gibbs phenomenon dominates the total error, with negligible contribution from SCAs. Only the magnitude of the real component of the residual is displayed.

The SCA magnitude and phase correction are small for most of the spectrum. The linear SCAs and the phase correction term each reach their maximum magnitude near the filter cutoff wavenumbers. This behavior is expected because the linear SCA term is greatest where the derivative of the spectrum difference is largest. The SCAs in Figure 6a have a peak magnitude of approximately 10% of the ZPD spectrum magnitude. However, after multiplication by the imaginary component of the phase correction shown in Figure 6b, the resulting peak magnitude is less than 0.1% the ZPD spectrum magnitude. This indicates that linear SCAs have a negligible effect on the measured spectrum. Error resulting from the Gibbs phenomenon, caused by interferogram truncation, is larger than the error associated with the linear SCAs. Figure 7 compares various error sources for linear scene change.

Linear SCAs have a relatively small effect on the real component of the spectrum. Even the largest SCA spikes near the notch filter cutoff wavenumber are small relative to the Gibbs phenomenon, which peaks in the same region. The interferogram-offset SCAs after SOC are even smaller. Table 2 compares these error sources for the same conditions as Table 1.

Errors from linear SCAs and interferogram-offset SCAs after SOC are smaller than the Gibbs phenomenon error. Linear SCA error does not depend on bandwidth. Linear SCA error is dependent on the transmission of the notch filter. This behavior is expected because the transmission affects the derivative magnitude while bandwidth does not. These results indicate that, with SOC applied, neither interferogram-offset SCAs nor linear SCAs constitute the dominant error source.

### 2.5. Quadratic SCAs

The linear SCAs and interferogram-offset SCAs after SOC have minimal effect on spectral accuracy. The final SCA term examined is the quadratic term in Equation (12). A quadratic scene change is simulated using the following scene function:

(24) $$s\left(x\right)={\left(\frac{x+\frac{x_{a}}{2}}{x_{a}}\right)}^{2}$$

**Figure 8:**
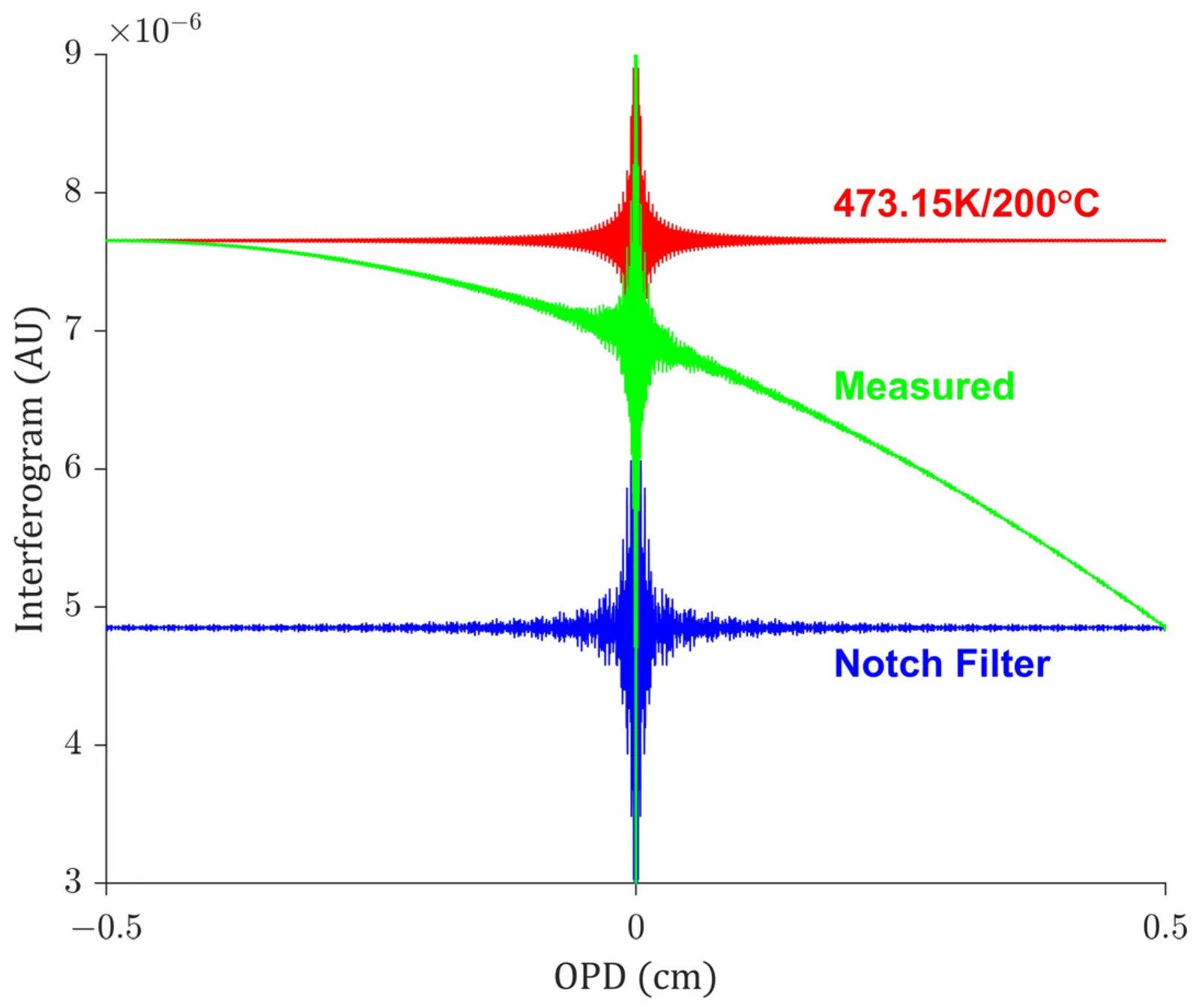
Simulated interferogram measurements for a quadratic scene change. The green curve represents the measured interferogram for the quadratic scene change. The red curve represents the measured interferogram for the unobscured blackbody, while the blue curve represents the measured interferogram for the blackbody observed through the notch filter.

Substituting Equation (24) into Equation (18) yields the measured interferogram for a simulated quadratic scene change. The measured interferogram is shown in Figure 8.

**Figure 9:**
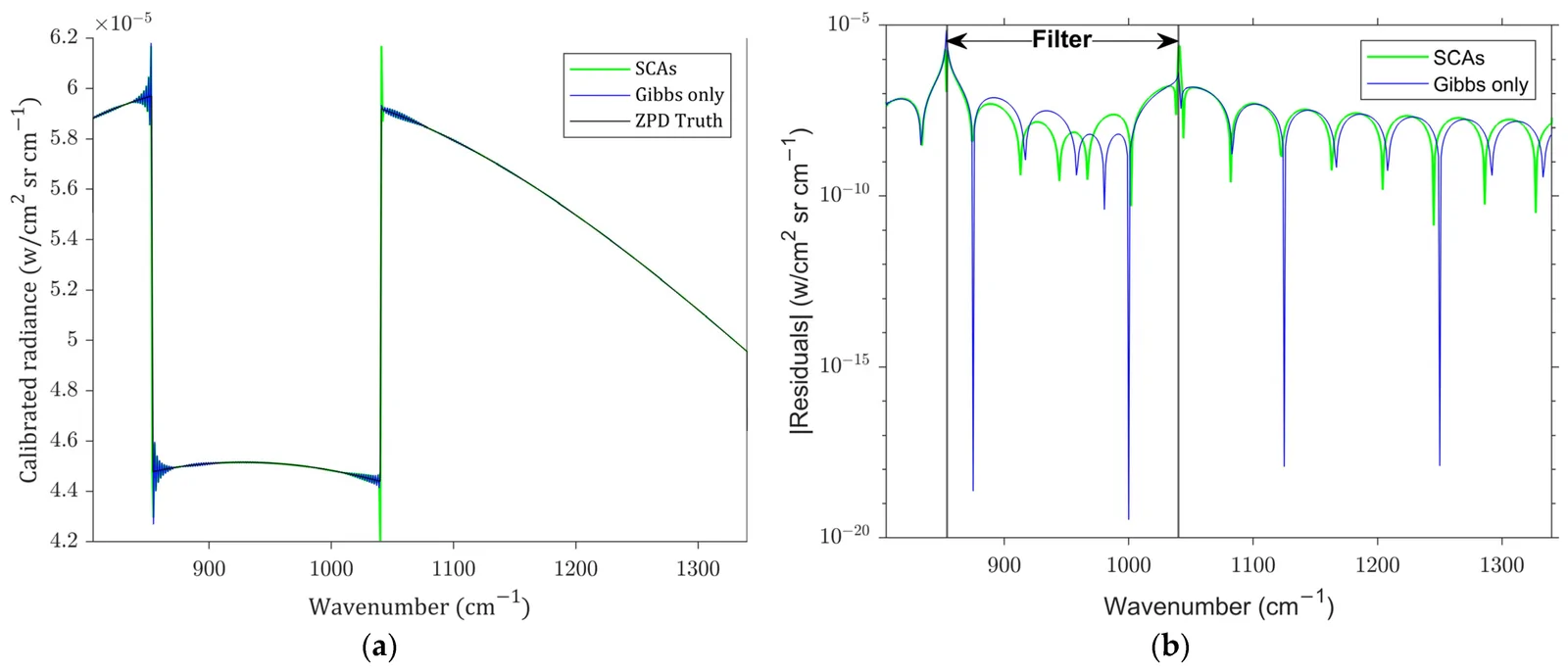
(**a**) Calibrated spectra for simulated quadratic scene change. The green curve represents quadradic SCAs superimposed on the ZPD spectrum. The blue curve represents Gibbs phenomenon superimposed on the ZPD spectrum. The black curve represents the truth spectrum observed at ZPD. (**b**) Comparison of error sources for simulated quadratic scene change. The green curve represents the error from quadratic SCAs, while the blue curve represents the error from Gibbs phenomenon. The notch filter cutoffs are indicated in black. Only the magnitude of the real component of the residual is displayed.

As in the linear case, SOC eliminates the interferogram-offset SCAs for the quadratic scene change. The scene function in Equation (24) produces a linear SCA term. Because the linear SCA term is imaginary, it does not affect the real component of the calibrated spectrum when phase correction is not applied. Only the quadratic SCAs remain. The quadratic SCAs have a similar magnitude to the Gibbs phenomenon, as shown in Figure 9.

The quadratic SCAs are similar in shape and magnitude to the Gibbs phenomenon, leading to increased error compared with Gibbs phenomenon alone. A comparison of these two error sources is presented in Table 3 for the conditions simulated in Table 1.

Quadratic SCAs produce errors that are smaller than, but comparable to, Gibbs phenomenon errors. Similar to linear SCAs, quadratic SCA errors do not depend on bandwidth but they do depend on transmission of the notch filter. These results indicate that quadratic SCAs contribute meaningfully to the error, but the Gibbs phenomenon is still the dominant source of error.

These simulation results provide an expectation for the subsequent experiment. Interferogram-offset SCAs occur throughout the spectrum and can be removed using SOC. After SOC, the interferogram-offset SCAs are negligible. Linear SCAs occur near sharp changes in the spectrum but appear in the imaginary component. Their effect on the real component is negligible. Lastly, quadratic SCAs are similar in appearance and slightly smaller in magnitude when compared to Gibbs phenomenon.

## 3. Materials and Methods

### Experimental Setup

**Figure 10:**
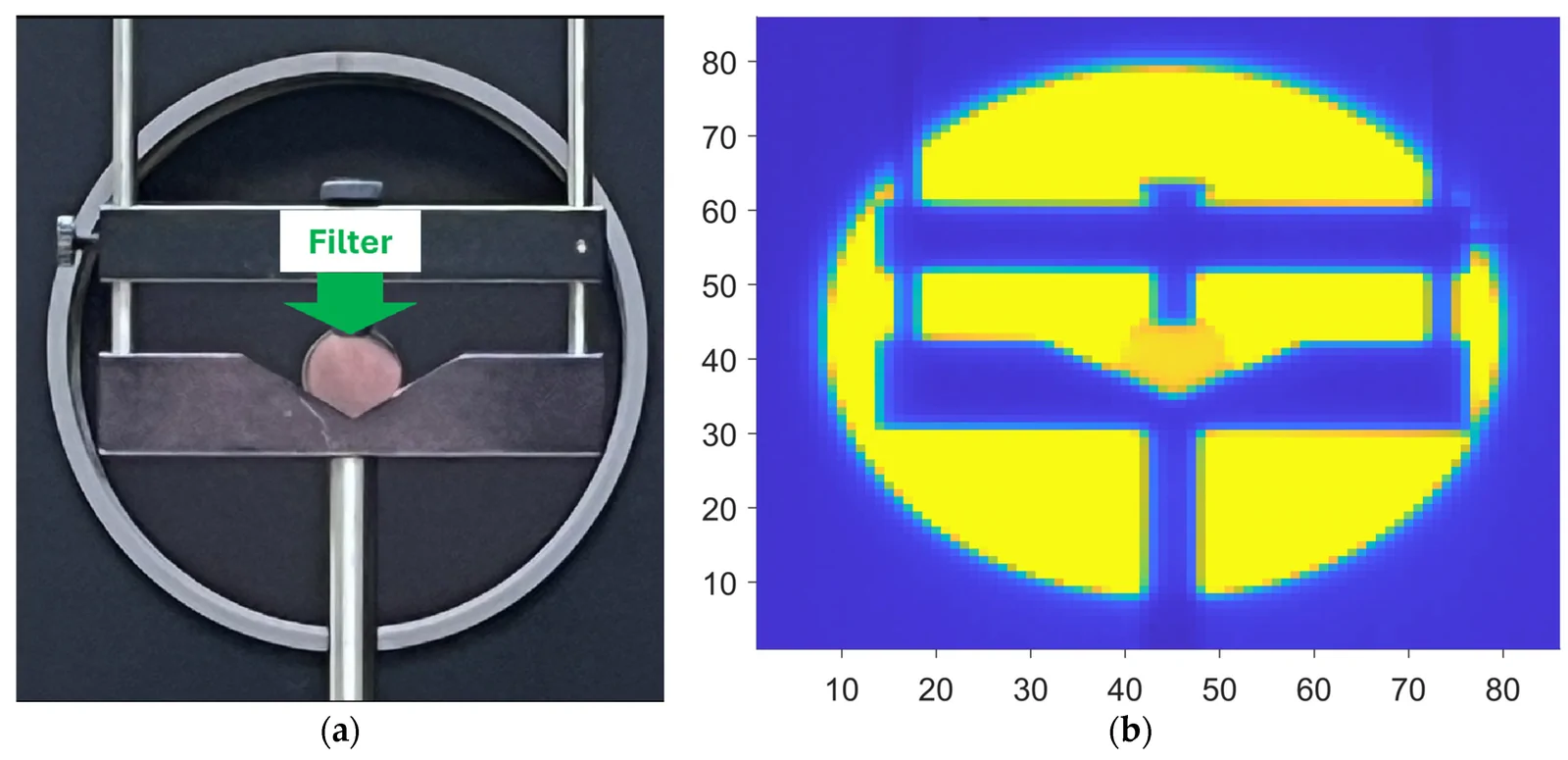
(**a**) Photograph of the experimental setup. The small circle in the holder is the notch filter. The larger circle in the background is the blackbody. The post at the bottom of the photograph is mounted on the travel stage. (**b**) Intensity image of the static notch filter in front of the blackbody. The large yellow circle is the blackbody, and the smaller orange circle near the center is the notch filter.

For these experiments, an FTS measured a blackbody while a notch filter moved through the field of view. The filter motion consisted of either constant velocity or constant acceleration. Section 2 predicts this movement will generate linear or quadratic SCAs in the measured spectra. A Telops hyper-cam mini xLW (Telops, Quebec City, QC, Canada), an FTS hyperspectral imager, was used to collect double-sided interferograms of static and moving targets [37]. Measurements were acquired with a spatial resolution of 320 × 256 pixels, a spectral resolution of 4 cm^−1^ corresponding to a MOPD of 0.15 cm, and a 25 μs integration time. A Fluke 4181 precision IR calibrator (Fluke, Everett, WA, USA) was placed on an optics table 2.75 m in front of the FTS. For the two-point calibration, the blackbody was set to 323 K and 523 K (50 °C and 250 °C), after which it was set to 473 K (200 °C) [38]. The blackbody was measured both unobscured and with the notch filter 18 cm in front of blackbody, as shown in Figure 10. Then, the notch filter was moved across the FOV with either a constant speed between 0.4 $\frac{mm}{s}$ and 20 $\frac{mm}{s}$ or a constant acceleration between 0.4 $\frac{mm}{s^{2}}$ and 5 $\frac{mm}{s^{2}}$.

The filter used was an Alluxa 10600 OD6 notch filter (Alluxa, Santa Rosa, CA, USA) [39]. The filter was mounted on a ThorLabs LTS150 long travel linear stage (Thor Labs, Newton, NJ, USA). Motion was controlled using Kinsesis motion control software (Version 1.1.4.59) [40,41]. Data collection was performed on 17 April 2026 in an indoor laboratory and required approximately 4 h. Atmospheric conditions were assumed to be constant throughout the experiment and were incorporated into the calibration [42].

## 4. Results

### 4.1. Linear Scene-Change Experimental Results

**Figure 11:**
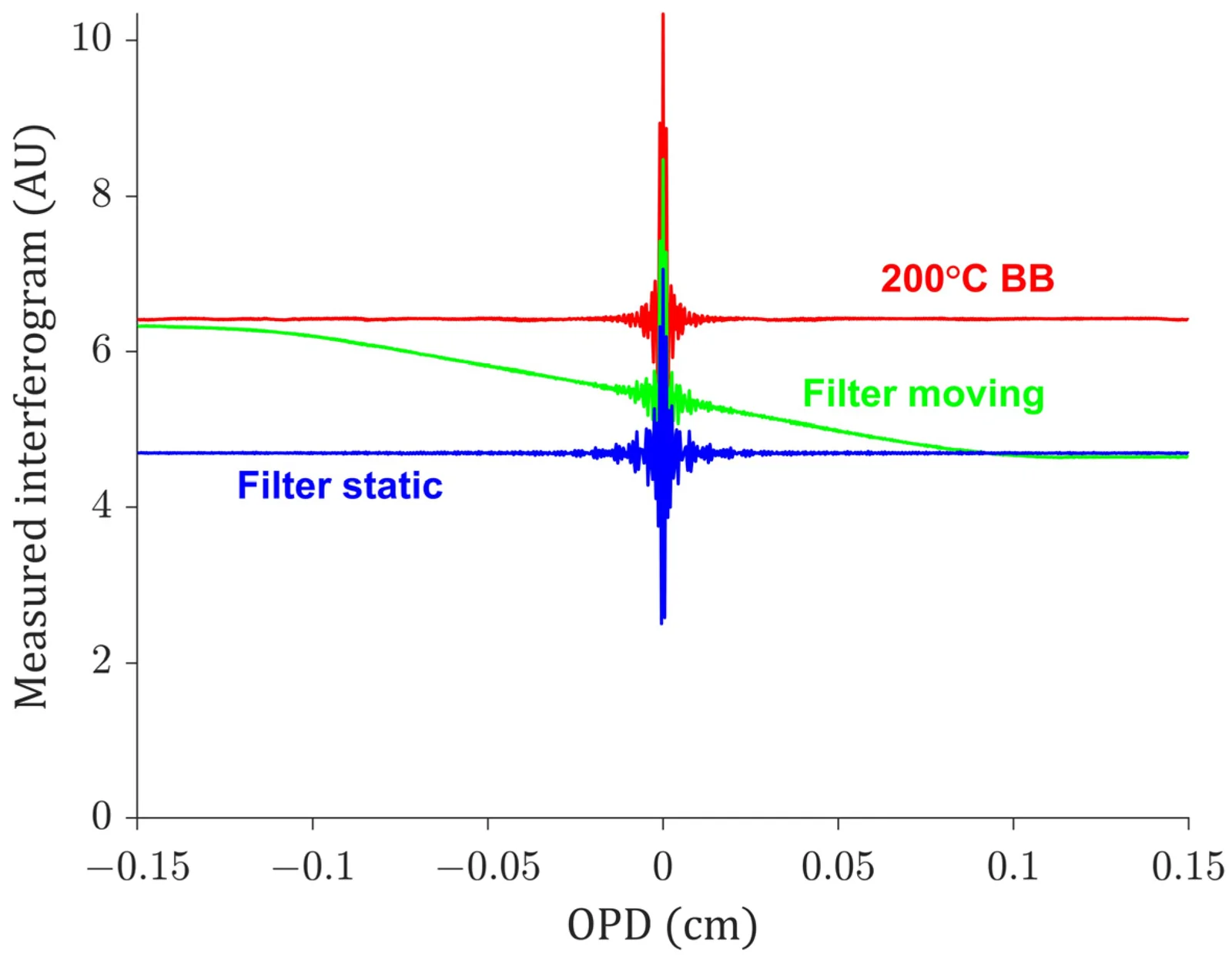
Measured interferogram associated with the linear scene change. The green curve represents the measured interferogram from the linear scene change. The red curve represents the measured interferogram from the unobscured blackbody. The blue curve represents the measured interferogram from the blackbody viewed through the static filter.

Beginning with the FOV fully filled by the unobscured blackbody, the filter transitioned across the FOV with constant speeds between 0.4 $\frac{mm}{s}$ and 20 $\frac{mm}{s}$. As the filter encompassed more of the FOV, the brightness and interferogram offset were reduced. For the instrument settings used, the 10 $\frac{mm}{s}$ velocity produced a transition on the same time scale as the interferogram collection, as shown in Figure 11.

**Figure 12:**
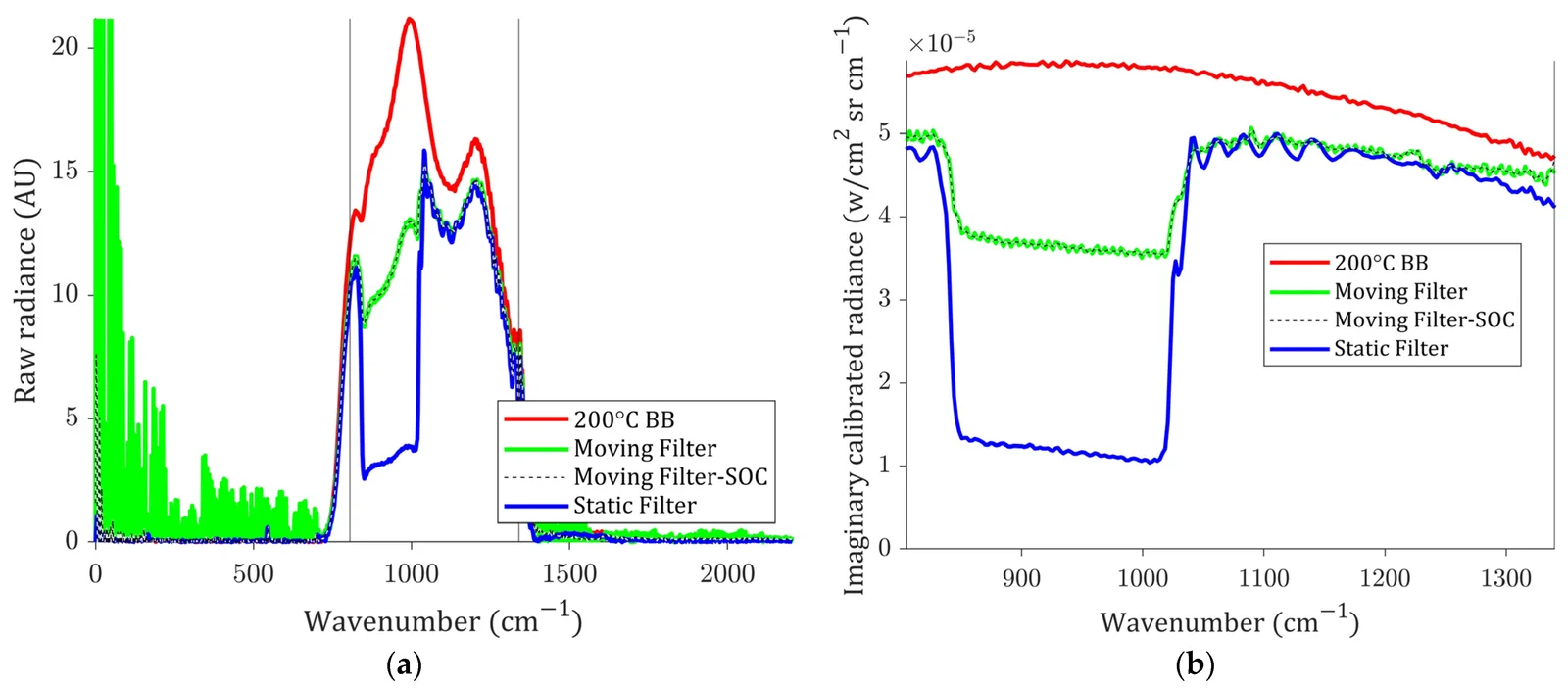
(**a**) Raw spectra with and without correction for a linear scene change. The green curve represents the raw spectrum with SCAs from the linear scene change using traditional processing. The black dashed curve represents raw spectrum from the linear scene change when processed with SOC. The red curve represents the raw spectrum of the unobscured blackbody. The blue curve represents the raw spectrum of the blackbody viewed through the static filter. (**b**) Calibrated spectra with and without correction. The green curve represents the calibrated spectrum with SCAs from the linear scene change using traditional processing. The black dashed curve represents the calibrated spectrum from the linear scene change when processed with SOC. The red curve represents the calibrated spectrum of the unobscured blackbody. The blue curve represents the calibrated spectrum of the blackbody viewed through the static filter. The moving filter curves represent the weighted average of the unobscured blackbody and the filtered blackbody, with the weights based on their respective fraction of the field of view.

Although the filter moved with constant velocity, the scene function was not perfectly linear. This behavior was expected because the first interferogram measurement is unlikely to coincide with the filter entering the sensor’s FOV. Similarly, the final interferogram measurement is unlikely to coincide with the filter fully filling the FOV. Additionally, diffraction effects may blur the filter boundary relative to the idealized scene change [20]. Traditional processing leaves SCAs both the raw and calibrated spectrum, as shown in Figure 12. The scene changes examined were relatively slow; therefore, a large SOC window of 0.09 cm was used for all experiments. SOC significantly reduced the interferogram-offset SCAs, as shown in Figure 12.

SCAs appear both inside and outside the instrument response region and increase in magnitude as the wavenumber approaches zero. This behavior is useful because signals outside the instrument response region primarily correspond to SCAs. Comparing signals outside and inside the instrument response region enables quantification of quantify SCAs in the absence of a truth spectrum. SCAs are quantified using signal-to-SCA ratio (SSR) defined as follows [7]:

(25) $$SSR=\frac{\frac{1}{N}\sum_{In}\left|Re\left[L_{raw}\left(v_{i}\right)\right]\right|}{\frac{1}{M}\sum_{Out}\left|Re\left[L_{raw}\left(v_{k}\right)\right]\right|}$$

The measured interferogram was processed using traditional methods and with SOC. Table 4 summarizes the improvement in SSR achieved with SOC for the linear scene change. For comparison the SSR and standard deviation of the static filter before and after SOC are also shown for the pixel rows that observed the transition.

SOC significantly improved SSR for the scene-change measurement and did not reduce SSR when no scene change was present when observing the static filter. These results demonstrate that SOC effectively eliminates interferogram-offset SCAs from linear scene changes with structured spectra, a result that has not been previously demonstrated.

A static filter without scene changes is provided for comparison. When no scene change is present noise and other effects still contribute to the out-of-band signal limiting the SSR upper bound. The largest non-SCA contributor to out-of-band is detector gain outside the instrument response region. This can be seen just to left of the instrument response region in Figure 12a.

**Figure 13:**
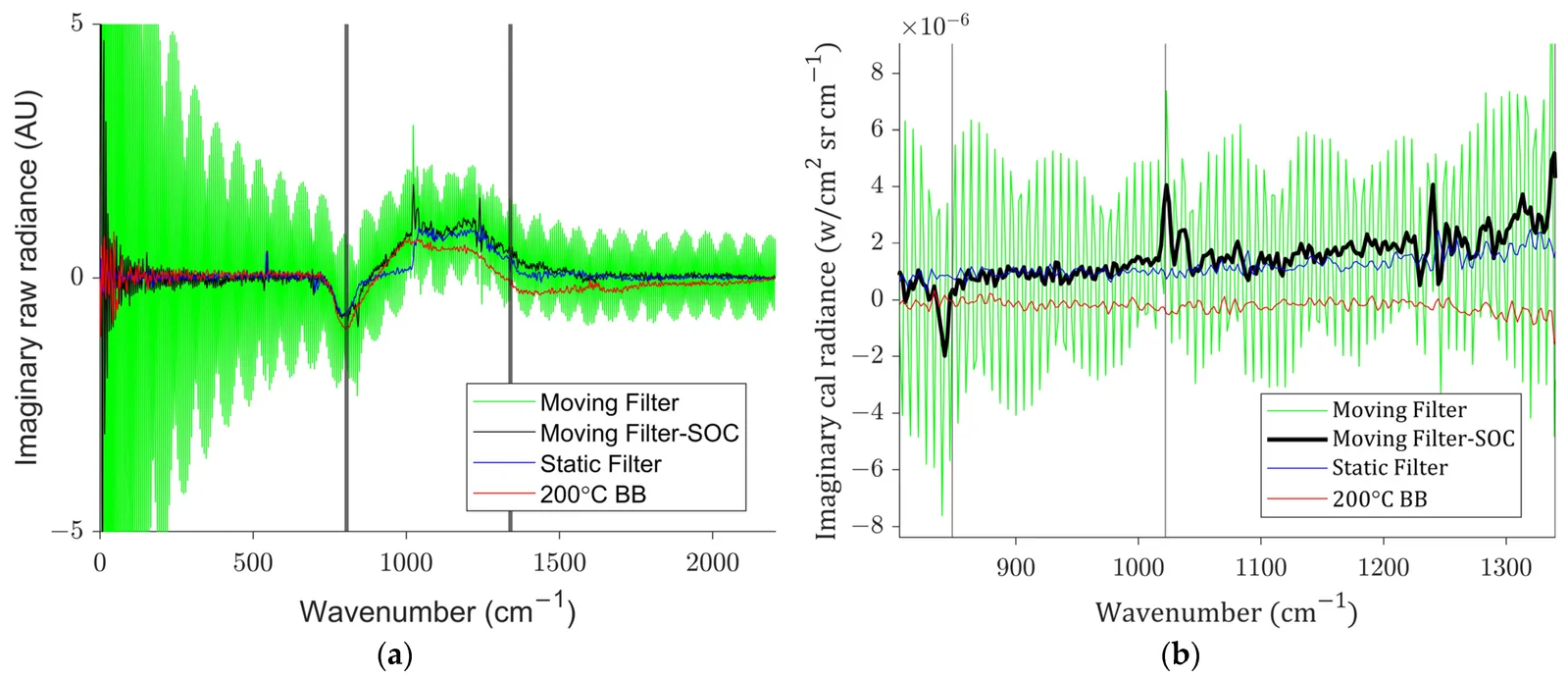
(**a**) Imaginary raw spectra; (**b**) imaginary calibrated spectra. The green curve represents the imaginary component of the linear scene-change spectrum from traditional processing. The black curve represents imaginary component of the linear scene-change spectrum when processed with SOC. The red curve represents the imaginary component of the unobscured blackbody spectrum. The blue curve represents imaginary component of the blackbody spectrum viewed through the static filter.

Interferogram-offset SCAs also appear in the imaginary component of the spectra. Fortunately, SOC removes the interferogram-offset SCAs appearing in the imaginary component of the spectrum alongside SCAs in the real component, as shown in Figure 13.

**Figure 14:**
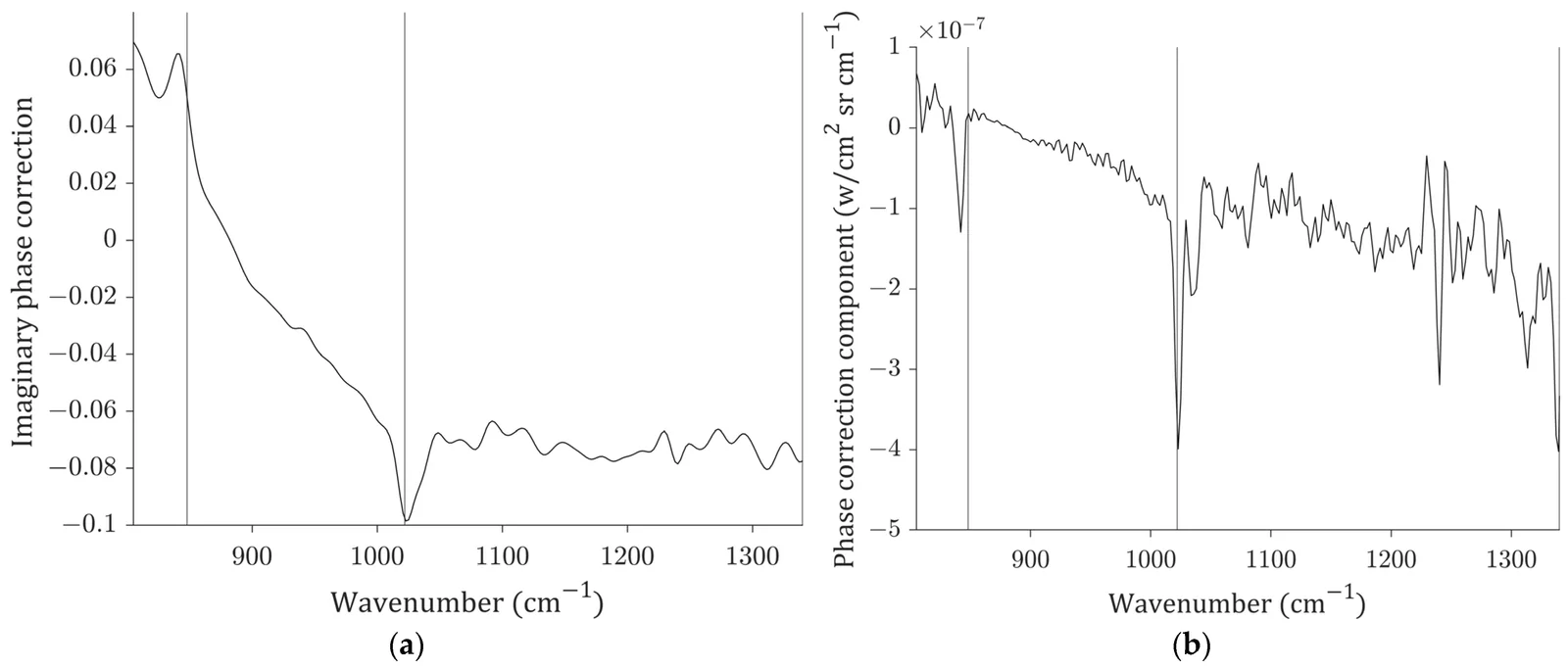
(**a**) Imaginary component of the phase correction. (**b**) The portion of the real component of the calibrated spectra which comes from phase correction. The black curve represents what is added to the real component of the calibrated spectrum and is calculated with the imaginary component of the spectrum and the imaginary component of the phase correction using Equation (23).

Linear SCAs have minimal effect on the real component of the spectrum. The effect can be determined using the imaginary component of the spectrum (Figure 13b) and phase correction (Figure 14a). Equation (23) is used to calculate the effect shown in Figure 14b.

The linear SCAs and the correction to phase errors in the instrument are combined in Figure 14b, giving a different appearance compared to Section 2.4, where the linear SCAs were examined independently. Despite this, the linear SCAs can still be seen near the notch filter’s cutoff wavenumbers. Each SCA spike appears as an absorption peak. This occurs even though one SCA spike appears as an emission peak in the imaginary component of the spectrum in Figure 13b. This behavior changes when the scene function is adjusted. If the blackbody starts obscured and then becomes unobscured, reversing the situation depicted in Figure 11, the SCA spikes appear as emission peaks rather than absorption peaks. Thus, some information about the scene function can be inferred from the SCA appearance. Although the SCA appearance may provide information about the scene change, similar information can be interpreted more easily from the measured interferogram shown in Figure 11.

Figure 14b shows a maximum SCA magnitude of approximately $4\times 10^{-7}\frac{w}{cm^{2}srcm^{-1}}$, whereas the calibrated spectrum in Figure 12b has a magnitude of approximately $4\times 10^{-5}$ $\frac{w}{cm^{2}srcm^{-1}}$. The SCAs have magnitudes of approximately 1% of the calibrated spectrum and occur near the notch filter cutoff wavenumbers. For the remainder of the calibrated spectrum, the linear SCAs are less than 1%. Although the experimental setup was designed to maximize the linear SCAs, their overall effect on the calibrated spectrum remains relatively small. These results indicate that linear SCAs have minimal effect on the calibrated spectrum.

### 4.2. Quadratic Scene-Change Experimental Results

**Figure 15:**
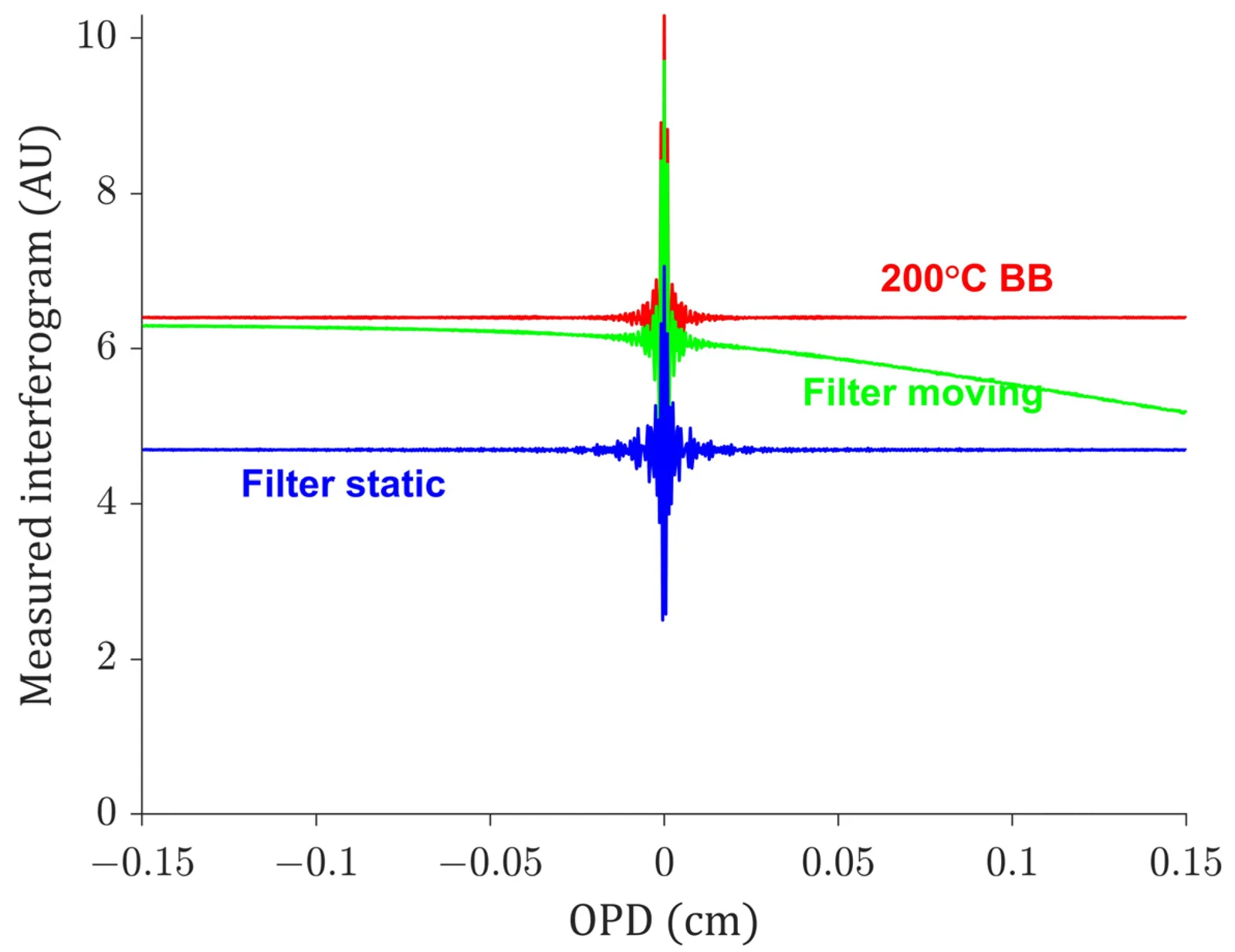
Measured interferogram associated with the quadratic scene change. The green curve represents the measured interferogram from the quadratic scene change. The red curve represents the measured interferogram from the unobscured blackbody. The blue curve represents the measured interferogram from the blackbody viewed through the static filter.

Section 2.4 and Section 2.5 suggest quadratic SCAs will have a greater effect than the linear SCAs seen in Section 4.1. To produce the quadratic scene change, the notch filter was moved across the FOV with a constant acceleration between 0.4 $\frac{mm}{s^{2}}$ and 5 $\frac{mm}{s^{2}}$. For the instrument settings used, the 0.4 $\frac{mm}{s^{2}}$ acceleration produced a quadratic transition on the same time scale as the interferogram collection, as shown in Figure 15.

Although the filter moved with constant acceleration, the scene function contained more than just the quadratic term. This behavior is similar to the scene with the linear scene-change results. The first interferogram measurement is unlikely to coincide with the filter entering the FOV. Similarly, the final interferogram measurement is unlikely to coincide with the filter fully filling the FOV. Furthermore, the filter did not have zero velocity at the first interferogram measurement. Even if these were all carefully controlled, diffraction effects blur the filter boundary relative to the idealized scene change. Fortunately, Section 4.1 demonstrated the linear term is expected to have negligible effect on the spectrum, and the interferogram-offset SCAs are removed with SOC.

The measured interferogram was processed using the traditional method and SOC. Table 5 summarizes the improvement in SSR achieved with SOC for the quadratic scene change. For comparison the SSR and standard deviation of the static filter before and after SOC are also shown for the pixel rows that observed the transition.

**Figure 16:**
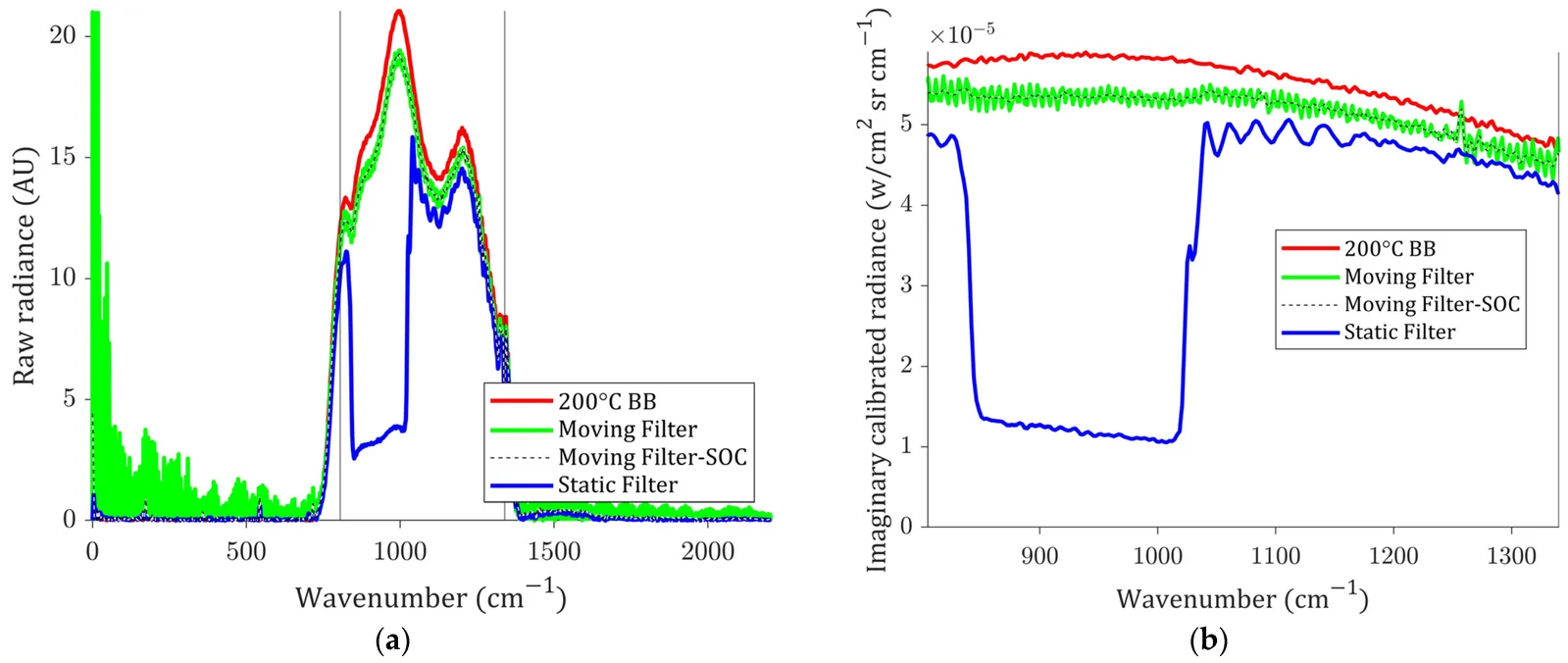
(**a**) Raw spectra with and without correction for a quadratic scene change. The green curve represents the raw spectrum with SCAs from the quadratic scene change using traditional processing. The black dashed curve represents raw spectrum from the quadratic scene change when processed with SOC. The red curve represents the raw spectrum of the unobscured blackbody. The blue curve represents the raw spectrum of the blackbody viewed through the static filter. (**b**) Calibrated spectra with and without correction. The green curve represents the calibrated spectrum with SCAs from the quadratic scene change using traditional processing. The black dashed curve represents the calibrated spectrum from the quadratic scene change when processed with SOC. The red curve represents the calibrated spectrum of the unobscured blackbody. The blue curve represents the calibrated spectrum of the blackbody viewed through the static filter. The moving filter curves represent the weighted average of the unobscured blackbody and the filtered blackbody, with the weights based on their respective fraction of the field of view.

As expected, SOC improved SSR in the scene-change case while maintaining SSR in the no scene-change case. Because the total signal at ZPD is higher for the quadratic case than for the static filter, the resulting SSR after SOC is also higher. These results demonstrate that SOC can eliminate interferogram-offset SCAs from quadratic scene changes with spectral structure, a result not previously demonstrated. The improvement in the raw and calibrated spectra is shown in Figure 16.

The quadratic SCAs are expected to peak near the notch filter’s cutoff wavenumbers. However, no evidence of such a peak is observed, suggesting that the SCAs are minimal. This result is unexpected because quadratic SCAs were anticipated to have a greater effect than the linear SCAs.

## 5. Discussion

**Figure 17:**
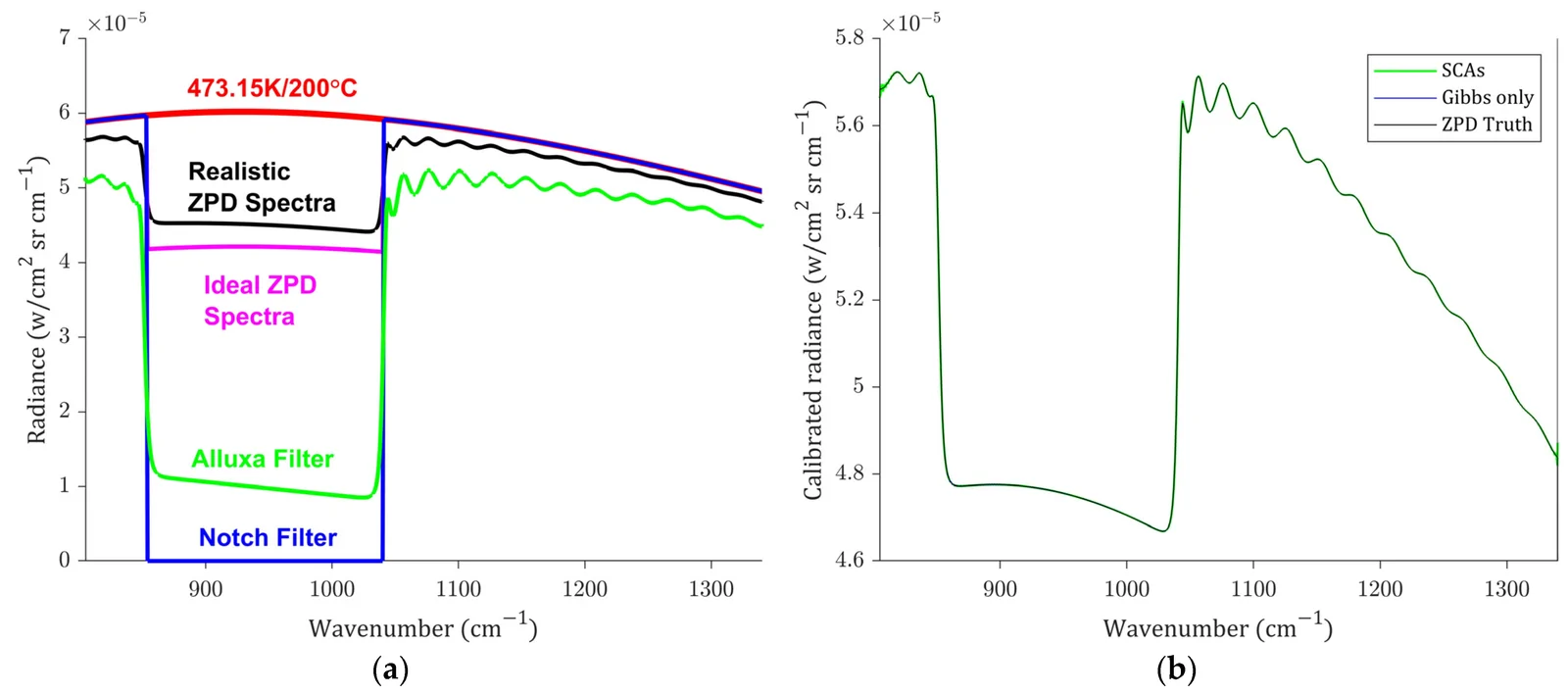
(**a**) Target spectra used in the simulation and experiment. The red curve represents the blackbody spectrum. The blue curve represents the blackbody spectrum viewed through an ideal notch filter. The green curve represents the blackbody spectrum viewed through the Alluxa notch filter. The black curve represents the spectrum observed at ZPD. At ZPD 70% of the FOV is filled by the blackbody and 30% is filled by the Alluxa filter. (**b**) Calibrated spectra obtained from the simulated quadratic scene change. The green curve represents the calibrated spectrum with quadratic SCAs. The blue curve represents the calibrated spectrum at ZPD without SCAs. The black curve represents the truth ZPD spectrum.

The experimental quadratic SCAs are smaller than those predicted by simulation. Several differences between the experimental setup and the simulation explain this discrepancy. The Alluxa notch filter used in the experiment has a transmission profile that differs from the ideal filter modeled in Section 2.4. The Alluxa filter exhibits a more gradual cutoff than the discontinuous filter assumed in the simulation. Next, the signal reaching the detector within the notch region is higher than predicted by the simulated. When the transmission is small, reflection and thermal emission contribute additional signal. Assuming the room and filter are both at 300 K (27 °C), the signal inside the notch approximates that of a room temperature blackbody rather than the zero signal assumed in the simulation. Lastly, the transmission outside the notch is less than one, reducing the signal from the hot blackbody. Together, these factors decrease the magnitude of the derivative near the cutoff wavenumber and reduce the associated SCAs. Figure 17a compares the idealized and experimentally realistic spectra.

When modeling this more realistic scenario, the largest quadratic SCA decreases to a magnitude of $9\times 10^{-8}\frac{w}{cm^{2}srcm^{-1}}$. This represents a significant decrease compared to the peak SCA magnitude of $2\times 10^{-6}\frac{w}{cm^{2}srcm^{-1}}$ predicted in Section 2.5. This updated SCA prediction corresponds to approximately 0.2% the magnitude of the spectrum in Figure 17b, compared to the previously predicted 10%. Thus, it is not surprising that SCAs were not detected in the quadratic scene-change experiment, given the revised SCA prediction. Furthermore, these results highlight the substantial difference between simulations assuming ideal behavior and those incorporating realistic experimental conditions. For realistic notch filter conditions, quadratic SCAs have negligible effect on the calibrated spectrum.

## 6. Conclusions

The following three types of SCAs were analyzed: interferogram-offset, linear and quadratic SCAs. Interferogram-offset SCAs were shown to be significant for linear and quadratic scene changes. Interferogram-offset SCAs can be mitigated using SOC. This mitigation was demonstrated for structured spectra under both linear and quadratic scene changes. These results expand the range of scenarios in which SOC has been shown to effectively mitigate interferogram-offset SCAs.

Linear SCAs were primarily limited to near the notch filter cutoff wavenumbers. Initially, they appear in the imaginary component of the spectrum. With phase correction, they can produce a small effect in the real component of the calibrated spectra.

Quadratic SCAs were shown to be negligible for the notch filter used in the experiment. However, when simulating an ideal filter, the error caused by quadratic SCAs is comparable to the error caused by the Gibbs phenomena. This suggests that there may be scenarios where quadratic SCAs are noticeable but difficult to distinguish from Gibbs oscillations.

Higher-order SCAs were not examined, but they are expected to have smaller magnitudes. This is due to the scaling factor preceding each SCA term decreasing and the increasing number of derivatives for successive terms. Furthermore, higher-order SCAs require the scene function to contain higher-order terms, which correspond to more complicated motion.

SCAs have been shown to be either easily correctable or negligible for FOV scene changes involving notch filters. Simulations suggest that sharp features may still generate SCAs during FOV scene changes. Absorption lines may be sharper than the notch filter used in this experiment. Because SCAs are larger near spectral transitions, they may affect atmospheric concentration estimates even when the broader radiometric accuracy is unaffected. Additionally, explosive scenes are commonly observed with FTS instruments but pose even greater difficulty. In an explosive scene, the temperature, plume size, and plume constituents each evolve rapidly. SOC has been applied to explosive data to mitigate interferogram-offset SCAs with promising results [8]. The lack of truth data makes it difficult to quantify the effects of higher-order SCAs in explosive scenarios. Although additional scenarios should be examined, the current evidence suggests that interferogram-offset SCAs can be mitigated using SOC and that higher-order SCAs are negligible for targets with smooth spectral features and potentially noticeable for targets with extremely sharp spectral features.

## Figures and Tables

**Table 1:** Error from interferogram-offset SCAs before and after SOC for different notch filters.

Notch Filter	Without SOC	With SOC
186 $cm^{-1}$ bandwidth	$3.3\times 10^{-2}$	$1.2\times 10^{-4}$
100 $cm^{-1}$ bandwidth	$1.8\times 10^{-2}$	$1.4\times 10^{-4}$
50 $cm^{-1}$ bandwidth	$9.0\times 10^{-3}$	$1.6\times 10^{-4}$
10 $cm^{-1}$ bandwidth	$1.8\times 10^{-2}$	$1.7\times 10^{-4}$
0.75 transmission	$2.4\times 10^{-2}$	$4.7\times 10^{-4}$
No scene change	$9.8\times 10^{-15}$	$4.9\times 10^{-4}$

**Table 2:** Simulated linear scene-change error comparison.

Notch Filter	Offset (With-SOC)	Linear	Gibbs
186 $cm^{-1}$ bandwidth	$1.2\times 10^{-4}$	1.9$\;\times 10^{-4}$	$2.4\times 10^{-2}$
100 $cm^{-1}$ bandwidth	$1.4\times 10^{-4}$	$1.9\times 10^{-4}$	$2.4\times 10^{-2}$
50 $cm^{-1}$ bandwidth	$1.6\times 10^{-4}$	1.9 $\times \;10^{-4}$	$2.6\times 10^{-2}$
10 $cm^{-1}$ bandwidth	$1.7\times 10^{-4}$	8.2 $\times \;10^{-5}$	$2.1\times 10^{-2}$
0.75 transmission	$4.7\times 10^{-4}$	$2.8\times 10^{-5}$	$1.7\times 10^{-2}$

**Table 3:** Simulated quadratic scene-change error comparison.

Notch Filter	Quadratic	Gibbs
186 $cm^{-1}$ bandwidth	$1.4\times 10^{-2}$	$2.3\times 10^{-2}$
100 $cm^{-1}$ bandwidth	$1.5\times 10^{-2}$	$2.3\times 10^{-2}$
50 $cm^{-1}$ bandwidth	$1.5\times 10^{-2}$	$2.6\times 10^{-2}$
10 $cm^{-1}$ bandwidth	$1.5\times 10^{-2}$	$2.1\times 10^{-2}$
0.75 transmission	$5.4\times 10^{-3}$	$8.7\times 10^{-3}$

**Table 4:** SSR with and without SOC for experimental linear scene change.

Scene Change	SSR Without SOC	SSR with SOC
Linear	3.8±0.4	24.3±0.7
Static filter	26.1±0.1	26.4±0.1

**Table 5:** SSR with and without SOC for experimental quadratic scene change.

Scene Change	SSR Without SOC	SSR with SOC
Quadratic	7±1	31.7±0.2
Static filter	26.1±0.1	26.4±0.1

## Data Availability

The datasets presented in this article are not readily available because they are not cleared for public release. Requests to access the datasets should be directed to Kody Wilson.

## Abbreviations

The following abbreviations are used in this manuscript:

FFT	Fast Fourier-transform
FOV	Field of view
FTS	Fourier-transform spectroscopy
IFFT	Inverse fast Fourier-transform
LOWESS	Locally weighted scatter plot smoothing
LWIR	Longwave infrared
NRMSE	Normalized root mean square error
OPD	Optical path difference
RCM	Recombobulate correction method
SCA	Scene-change artifact
SOC	Smooth offset correction
SSR	Signal-to-SCA ratio
ZPD	Zero-path difference
